# Strong field–driven subcycle band structure modulation and dephasing control

**DOI:** 10.1126/sciadv.aeg3966

**Published:** 2026-08-28

**Authors:** Francis Walz, Shashank Kumar, Amirali Sharifi Olounabadi, Yuyan Zhong, Russell Zimmerman, Siddhant Pandey, Eric Liu, Liang Z. Tan, Niranjan Shivaram

**Affiliations:** ^1^ Department of Physics and Astronomy, Purdue University, 525 Northwestern Ave., West Lafayette, IN 47907, USA.; ^2^ Molecular Foundry, Lawrence Berkeley National Laboratory, 1 Cyclotron Rd., Berkeley, CA 94720, USA.; ^3^ Purdue Quantum Science and Engineering Institute, 1205 W State St, West Lafayette, IN 47907, USA.

## Abstract

Over the past decade, ultrafast electron dynamics in the solid state have been extensively studied using various strong light-matter interaction techniques, such as high-harmonic generation. These studies lead to multiple interpretations of light-matter interaction in the strong-field regime, with exact mechanisms not yet fully understood. It is well known that strong-field interaction with a crystalline solid leads to substantial modification of its band structure and, hence, its optical properties on ultrafast timescales. In this work, we present measurements of ultrafast electric-field observables in magnesium oxide using a nonresonant nonlinear optical interaction. Using field observables, we show that strong laser fields modulate the band structure on subcycle timescales, thereby altering the material’s nonlinear optical response. We perform time-dependent perturbation theory calculations using a field-dependent dispersion relation and semiconductor Bloch equation calculations, both of which agree with experimental observations. Furthermore, we extract pulse decay times from the real-time signal electric field envelope and show subcycle control of dephasing times. Our work offers a new perspective on strong field–driven electron dynamics in solids through electric-field observables. The demonstrated attosecond modulation of the nonlinear response could have important implications for quantum light generation and quantum spectroscopy using nonlinear optical processes.

## INTRODUCTION

The interaction of strong, ultrafast laser pulses with solids can give rise to many intriguing phenomena that have been extensively studied using multiple ultrafast techniques. Examples include high-harmonic generation (HHG) (*1*, *2*), the realization of the Haldane model in a laser-dressed crystal (*3*), and transient optical response modulation, also known as the dynamical Franz-Keldysh effect (*4*–*7*). These effects are fundamental to understanding electron dynamics in the strong field regime. Several theoretical models have been developed to study these phenomena, such as the Houston basis formalism (*8*, *9*), Wannier functions (*10*, *11*), and the semiconductor Bloch equation (SBE) (*12*–*14*) to model HHG in solids. To understand the dynamical Franz-Keldysh effect, more sophisticated time-dependent density functional theory techniques have been used (*6*, *15*). Despite differences in approach, these models inherently incorporate nonperturbative strong-field effects in the material to gain insight into ultrafast electron dynamics, as measured by various experimental observables.

In the study presented here, we use ultrafast nonlinear optical spectroscopy with electric-field measurement of the four-wave mixing (FWM) signal to probe the effects of strong field–driven electron dynamics on the nonlinear response of a solid. FWM is a third-order nonlinear process in which three coherent femtosecond pulses interact with a crystalline solid to generate a fourth pulse whose amplitude is proportional to the material’s third-order susceptibility ($\chi^{\left(3\right)}$) (*16*). Whereas most strong-field studies in solids have been carried out at high intensities where HHG is efficient (*1*, *2*), here, we work at lower intensities (∼0.1 TW/cm^2^), below typical intensities used in HHG experiments, where electron-hole pairs are not driven across the Brillouin zone yet nonperturbative effects remain substantial. Measuring the complete electric field of the nonlinear signal (temporal amplitude and phase) provides direct access to subcycle modulations of $\chi^{\left(3\right)}$ through field observables such as the temporal chirp, which are not available in intensity- or spectrally resolved measurements. The temporal phase, in particular, is highly sensitive to small field–driven modifications of the response function and also encodes the dephasing of the third-order coherence through the temporal envelope of the emitted signal field.

Prior FWM studies in solids have focused largely on resonant interactions in quantum wells and quantum dots (*17*–*20*), where excitonic dynamics dominate and propagation effects in optically thick samples introduce additional complications (*21*–*23*). Recent advances in free-electron lasers have extended FWM into the extreme ultraviolet (*24*, *25*), and nonlinear response functions have been inferred from HHG in solids (*26*). Building on earlier electric-field measurements of FWM in molecular gases (*27*, *28*), here, we apply the approach to a wide-gap crystal under strong-field conditions to investigate the strong-field modification of the nonlinear response function.

We measure electric-field observables for degenerate FWM (DFWM) in magnesium oxide (MgO) to study the strong-field modification of the band structure and, hence, the nonlinear response function in the material. Using the TADPOLE technique (*29*), the amplitude and phase of the DFWM output field are measured, providing complete information about the ultrafast nonlinear light-matter interaction. We also develop a theoretical model on the basis of time-dependent perturbation theory, combined with a field-dependent dispersion relation (*30*), to simulate the DFWM process in a large-bandgap crystal. We study the modulation of the amplitude and the temporal chirp of the output signal as we vary the time delay between incident pulses with attosecond resolution. We find that both the amplitude and chirp oscillate with time delay, and our model, with good agreement, predicts that this oscillation can occur because of ultrafast modulation of the band structure of MgO under strong electric fields. We additionally model our experiment using SBEs and find good agreement with our experimental results. We then demonstrate that the microscopic nonlinear response function acquires a field-dependent correction, which accounts for the observed oscillations. Last, we extract pulse decay times representing third-order coherence dephasing times from our measured signal electric field (*31*) and demonstrate subcycle control of dephasing time resulting from the strong field–driven modulation of the band structure.

## RESULTS AND DISCUSSION

### Amplitude and phase of the measured DFWM electric field

**Fig. 1.**
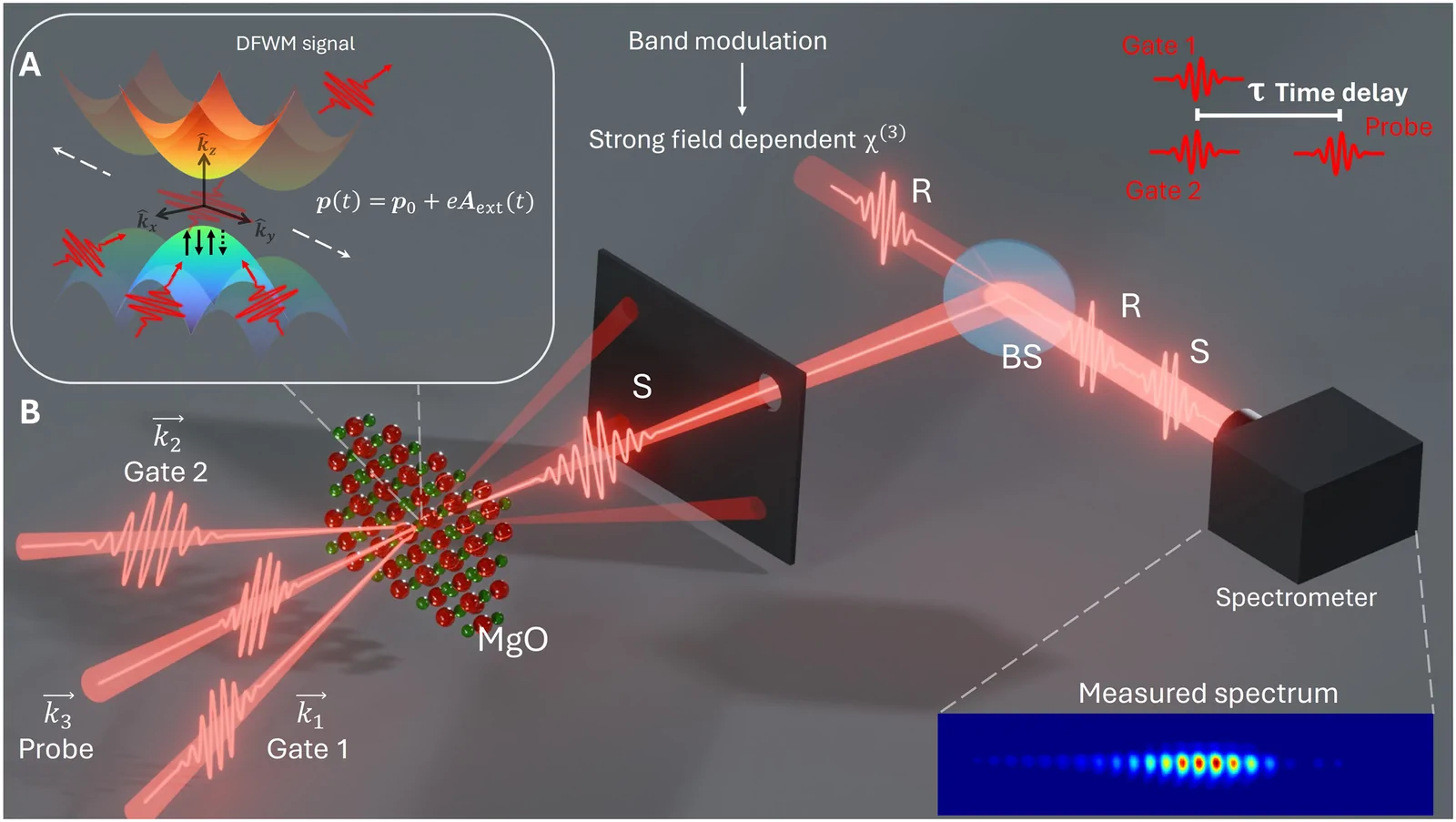
Ultrafast strong field–driven band modulation experiment. (**A**) The net electric field resulting from the superposition of three femtosecond pulses interacts with MgO and substantially modifies the band structure of the material by making the dispersion relation field-dependent. This interaction modulates the band structure (represented by opaque curves) and shifts it (transparent curves) from its original position on an ultrafast timescale. The measured output electric field from an FWM nonlinear process can capture this modulation, providing insights into the dynamics of strong-field interactions in a solid. (**B**) Schematic diagram of the spectral interferometry measurement for DFWM: The experimental setup illustrates the interaction of three ultrafast pulses (Gate 1, Gate 2, and Probe) with the MgO crystal and the generation of a DFWM Signal pulse. This signal interferes with a Reference pulse at a spectrometer, resulting in a spectral interference measurement. The two Gate pulses are phase locked and delayed with respect to the Probe pulse. R, Reference pulse; S, Signal pulse; BS, beam splitter.

**Fig. 2.**
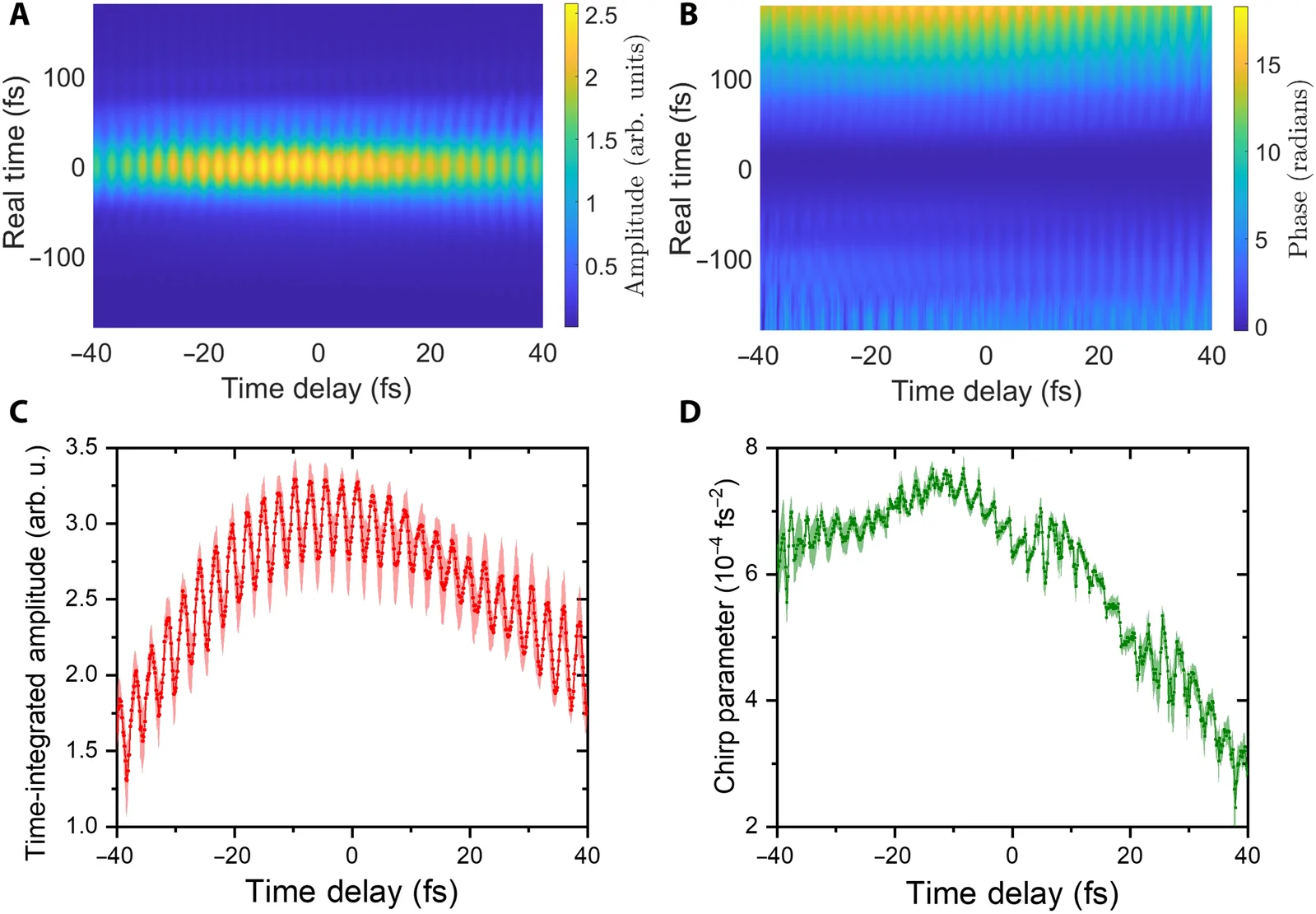
Experimentally measured amplitude and phase. (**A**) Amplitude $|E_{0}\left(t,\tau \right)|$ and (**B**) phase φ(t,τ) of the DFWM signal in MgO. The *y* axis corresponds to real time in the frame of the pulse, and the *x* axis signifies the time delay between the Gate and Probe pulses. (**C**) Time-integrated temporal amplitude and (**D**) temporal chirp as a function of time delay τ with a delay step of 200 as extracted from (A) and (B), respectively. The shaded region represents the standard error in the measurement obtained from eight different time delay scans.

Our experiment uses the spectral interferometry–based technique of TADPOLE (*29*) to completely characterize the DFWM signal generated using three 50-fs near-infrared (NIR) pulses centered around 800 nm ($\hbar \omega_{0}\approx 1.55$ eV). These three linearly polarized pulses interact with the MgO crystal with an intensity of ∼0.1 TW/cm^2^ in a BOXCAR configuration (*32*), producing a fourth beam in a new direction that satisfies the phase matching condition $k_{s}=k_{1}-k_{2}+k_{3}$. Figure 1A illustrates the schematic representation of the highest valence band and the lowest conduction band of MgO, along with the FWM process occurring within the crystal. This FWM process occurs with a simultaneous modulation of the band resulting from the net electric field present during the interaction, as discussed later. Given that the MgO bandgap is about 7.8 eV and the photon energy of each incoming pulse is 1.55 eV, the interaction is highly off-resonant. A reference beam then interferes with the output signal, and spectral interference is measured using a spectrometer. Representative interference fringes from the measurement along with the experimental setup are shown in Fig. 1B. From this interference, we can extract the temporal amplitude and phase of the electric field of the DFWM signal (more details in Materials and Methods). The electric field is then measured for each time delay (τ) between two phase-locked Gate pulses and the Probe pulse, with a time delay resolution of 200 as. Therefore, the complete electric field of the DFWM signal can be expressed as $E\left(t,\tau \right)=|E_{0}\left(t,\tau \right)|\text{exp}\left[-i\varphi \left(t,\tau \right)\right]$, where the temporal amplitude $|E_{0}\left(t,\tau \right)|$ and phase φ(t,τ) depend on real time *t* and the time delay between the Gate and Probe pulses τ. Both the amplitude and phase are plotted in Fig. 2 (A and B), respectively. The temporal phase of a femtosecond pulse (see Materials and Methods for the temporal phase extracted from the DFWM signal) is typically expanded as a Taylor series in time as

$$\varphi \left(t,\tau \right)=\varphi_{0}+a\left(\tau \right)t+b\left(\tau \right)t^{2}+c\left(\tau \right)t^{3}+d\left(\tau \right)t^{4}+e\left(\tau \right)t^{5}$$ (1)

The second-order coefficient b(τ) associated with the temporal chirp is extracted as a function of the time delay τ. We found that for polynomial orders beyond the fifth order, improvements in the goodness of fit become negligible, and thus, the polynomial is truncated at the fifth order. We integrate the extracted amplitude over the total pulse duration for each time delay; this is called the time-integrated amplitude and is defined as $\int |E_{0}\left(t,\tau \right)|\mathrm{dt}$. The time-integrated amplitude as a function of time delay is shown in Fig. 2C. Figure 2D shows the chirp parameter b(τ) as a function of the time delay with attosecond resolution. The shaded area represents the standard error obtained from eight different time delay scans in the experiment. Modulation of nonlinear signals has previously been observed in semiconductors, where a strong NIR pump pulse generates mid-infrared sidebands by mixing with a strong terahertz pulse (*33*). The time delay between the two mixing pulses modulates the spectral intensity of the mid-infrared sideband. However, subcycle modulation in electric field observables, as shown here, has not been observed previously.

### Strong field–driven nonlinear response modulation

The output DFWM signal depends on the third-order polarization of the crystal. The spectrum of third-order polarization can be written in terms of the nonlinear response function, i.e., third-order susceptibility $\chi^{\left(3\right)}\left(-\omega ;\omega ,-\omega ,\omega \right)$, which is a material property. However, at higher intensities, higher-order contributions can arise. Most generally, the polarization is (*16*)

$$\begin{array}{c} P_{i}\left(\omega \right)=\chi_{\mathrm{ij}}^{\left(1\right)}{\tilde{E}}_{j}\left(\omega \right)+\chi_{\text{ijkl}}^{\left(3\right)}{\tilde{E}}_{j}\left(\omega \right){\tilde{E}}_{k}\left(\omega \right){\tilde{E}}_{l}\left(\omega \right)\\ +\chi_{\text{ijklmn}}^{\left(5\right)}{\tilde{E}}_{j}\left(\omega \right){\tilde{E}}_{k}\left(\omega \right){\tilde{E}}_{l}\left(\omega \right){\tilde{E}}_{m}\left(\omega \right){\tilde{E}}_{n}\left(\omega \right)+\cdots \end{array}$$ (2)

If the polarization vectors of the input and output electric fields are in the same direction, as in our experiment, the indices i,j,k,⋯ are the same. For the remainder of this discussion, we omit the polarization subscripts for brevity. Here, $\tilde{E}\left(\omega \right)=\left[{\tilde{E}}_{1}\left(\omega \right)e^{ik_{1}\cdot r}+{\tilde{E}}_{2}\left(\omega \right)e^{ik_{2}\cdot r}+\right.\left.{\tilde{E}}_{3}\left(\omega \right)e^{ik_{3}\cdot r}+c.c.\right]$. On the basis of the phase-matching condition mentioned above, the only terms that contribute to the measured signal are

$$\begin{array}{l} P_{\text{DFWM}}=\chi_{0}^{\left(3\right)}{\tilde{E}}_{1}\left(\omega \right){\tilde{E}}_{2}^{*}\left(\omega \right){\tilde{E}}_{3}\left(\omega \right)e^{i\left(k_{1}-k_{2}+k_{3}\right)\cdot r}\\ +\chi_{0}^{\left(5\right)}{\tilde{E}}_{1}\left(\omega \right){\tilde{E}}_{2}^{*}\left(\omega \right){\tilde{E}}_{3}\left(\omega \right)\left({|{\tilde{E}}_{1}|}^{2}+{|{\tilde{E}}_{2}|}^{2}+{|{\tilde{E}}_{3}|}^{2}\right)e^{i\left(k_{1}-k_{2}+k_{3}\right)\cdot r}+\cdots \end{array}$$ (3)

Here, ${\tilde{E}}_{1},{\tilde{E}}_{2}$, and ${\tilde{E}}_{3}$ are the two Gate and Probe pulse electric fields in the frequency domain, respectively. The introduction of a time delay τ between Gate and Probe pulses will result in a phase term of exp(−iωτ) in the electric field of the Gate pulses. However, this phase term does not result in a τ-dependent modulation of the amplitude or phase of the polarization corresponding to the DFWM process when $\chi_{0}^{\left(3\right)}$ is independent of the input fields (for a detailed calculation, see the Supplementary Materials). In the presence of moderately strong fields, nonlinear response functions are expected to be substantially modified as observed, for example, in studies of the dynamical Franz-Keldysh effect using attosecond transient absorption spectroscopy (*6*, *33*). Thus, we model the attosecond modulation of the nonlinear response function resulting from strong field interaction such that $\chi^{\left(3\right)}$ becomes an instantaneous function of the incoming electric fields, $\chi^{\left(3\right)}\to \chi^{\left(3\right)}\left(\tilde{E}\right)$. The derivation of the microscopic $\chi^{\left(3\right)}$ dependence on the strong field is provided in the Supplementary Materials and will be discussed in detail later in this section.

In high-intensity laser fields, excited electron-hole pairs can extend beyond the edge of the Brillouin zone, leading to Landau-Dykhne–type transitions. Such effects have been observed in high-harmonic spectra (*34*, *35*). These transitions provide a powerful means to probe modifications of the band structure induced by intense laser pulses. To directly probe band-structure modifications, time- and angle-resolved photoelectron spectroscopy (Tr-ARPES) can be used, as it images electron dynamics in materials with simultaneous momentum and energy resolution. Ab initio simulations have also been performed to study Tr-ARPES in periodic systems under strong fields (*36*). Most recently, a Tr-ARPES experiment on bismuth telluride observed field-dressed bands in the presence of an intense laser field (*37*). This suggests that the ultrafast modification of the band structure (or adiabatic field-dressed bands) offers a useful framework for understanding off-resonant electron interactions in the strong-field regime. Alternative perspectives for interpreting these field-dressed bands have also been proposed, most notably within the framework of Floquet theory (*38*). While band structures can also be engineered through alternative routes such as strain (*39*), doping (*40*), or heterostructure design (*41*), the present work focuses exclusively on the dynamic, laser-driven modulation of the band structure (*30*).

For a rigorous quantitative analysis, we start with time-dependent perturbation theory to simulate the DFWM experiment. However, the time-integrated amplitude and the chirp of the calculated signal electric field do not exhibit any of the oscillatory features observed in the experiment (see fig. S6, red versus blue curves). This aligns with the expectation that the field-free response function accounts only for perturbative wave-mixing processes (*16*, *42*) and cannot include any field-driven modification of the material properties.

To incorporate nonperturbative effects, we use an ultrafast band-modulation model (*30*). In the presence of an external field, the crystal momentum of the electrons can be written as $p\left(t\right)=p_{0}+eA_{\text{ext}}\left(t\right)$ (*2*, *8*, *9*, *30*, *43*), where $p_{0}$ denotes the field-free crystal momentum, and $A_{\text{ext}}\left(t\right)$ is the vector potential of the external field. This results in the time-dependent modification of the electronic dispersion relation, i.e., $\epsilon \left(p\right)\to \epsilon \left[p_{0}+eA_{\text{ext}}\left(t\right)\right]$, where ϵ is the energy of the electron. The details of the calculations are provided in the Supplementary Materials. A theoretical investigation of the nonlinear optical response under strong light-matter interaction in MgO has previously been carried out using the Floquet-Bloch formalism (*44*). Here, we demonstrate that incorporating nonperturbative effects (in particular, band-structure modulation) within a perturbative framework yields excellent agreement with the experiment.

Two remarks on this construction are in order. The band modulation is treated in the velocity gauge, in which the band energies become instantaneous functions of the field; the equivalent description in the length gauge corresponds to Bloch oscillations of electrons in the valence and conduction bands (*12*), the two pictures being related by a gauge transformation (*45*, *46*). The velocity-gauge formulation is adopted here as it is more suitable for interpreting the observed subcycle oscillations in field observables. Furthermore, the validity of embedding a nonperturbative effect within a perturbative response function depends on the experimental regime. The modest intensities and far off-resonant conditions used in our experiment result in light-matter energy transfer that is predominantly reversible, and real conduction band population transfer remains small (see fig. S7A in the Supplementary Materials) (*47*). The interband coherence underlying the third-order response is therefore dominantly virtual, while the strong-field correction to the response function arises from coherent dressing of the bands rather than from field-induced changes in real carrier population.

**Fig. 3.**
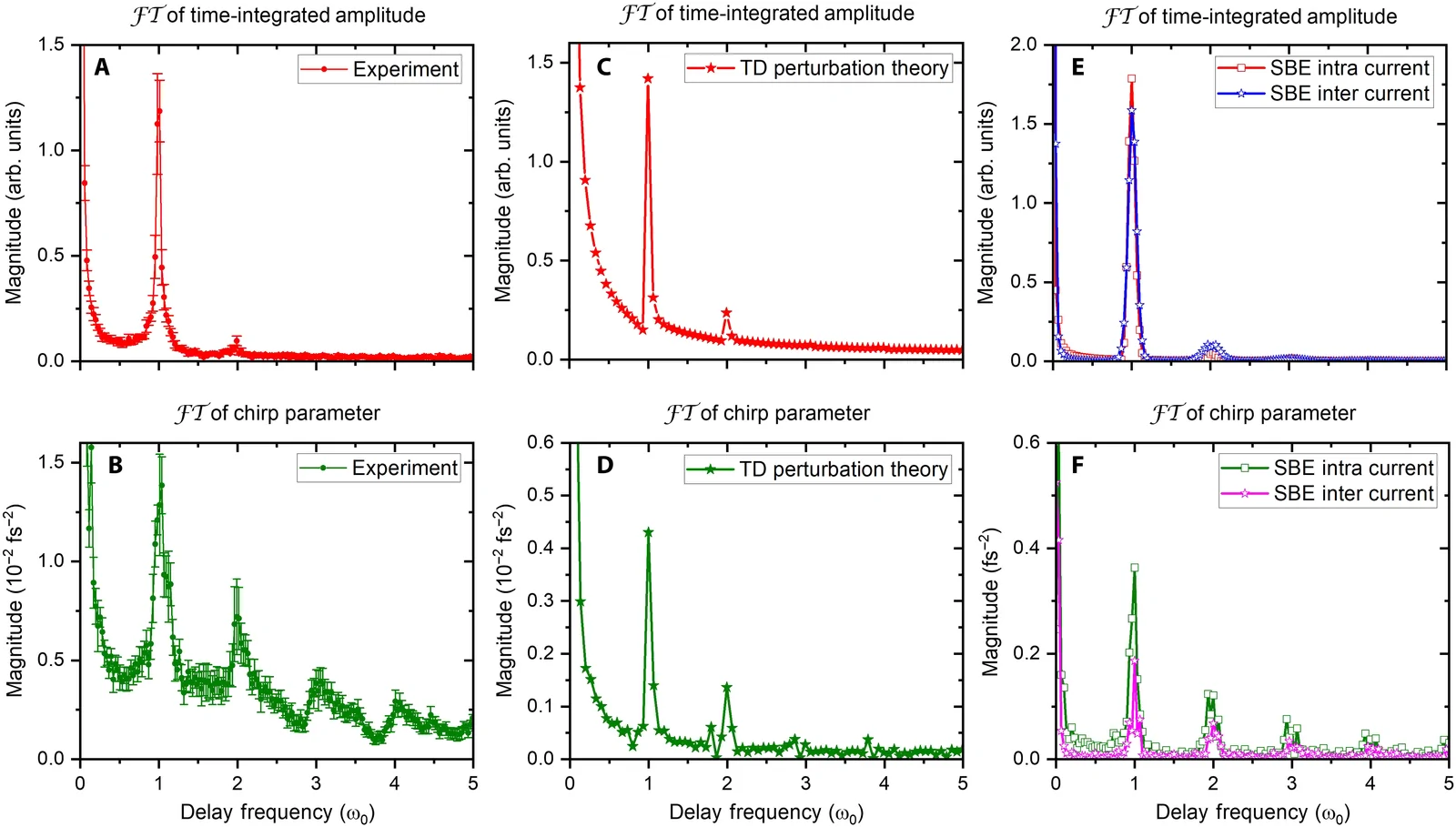
Fourier transform in time delay. The Fourier transforms (FT) with respect to the time delay of the experimentally measured (**A**) time-integrated amplitude and (**B**) temporal chirp parameter are shown. These results are compared with the corresponding (**C**) time-integrated amplitude and (**D**) chirp parameter obtained from the electric field calculated using time-dependent (TD) perturbation theory, which includes the band-structure modulation induced by the strong field (see the main text for details). The calculation parameters are as follows: IR pulse intensity ($I_{0}=0.2\;\text{TW}/{\text{cm}}^{2}$), photon energy ($\hbar \omega_{0}=1.55\;\text{eV}$), and MgO bandgap (Δ=7.78 eV). The (**E**) time-integrated amplitude and (**F**) temporal chirp are also extracted from the calculated output electric field using the SBEs, where the contributions from intra- and interband currents are shown separately.

We perform a Fourier transformation (FT) of our field observables in the time-delay domain. Figure 3 (A and B) shows the FT of the time-integrated amplitude and the chirp of the measured DFWM electric field shown in Fig. 2, respectively. We emphasize that the FT is in the time-delay τ domain and not in real time *t*, and the observed frequency components reflect delay-dependent modulations. We compare these with the theoretically calculated field observables after the inclusion of ultrafast modulation of the band structure within time-dependent perturbation theory in Fig. 3 (C and D). The calculation shows good agreement with the experimentally measured field observables for an input intensity of 0.2 TW/cm^2^, which is close to the experimentally measured intensity of ∼0.1 TW/cm^2^. The uncertainty in experimentally measured intensity is typically large for femtosecond pulses because of a complex beam profile at the focal spot. The time-integrated amplitude exhibits a strong $1\omega_{0}$ component of the delay frequency ($\omega_{0}$ is the central frequency of the NIR pulse). This is because the amplitude of the net incoming electric field modulates with time delay between the Gate and Probe pulses. As a result, the nonlinear response function is modulated via adiabatic field-dressed bands (see section S2 for details). The chirp, as shown in Fig. 3 (B and D), is more sensitive to such modulation of the nonlinear response function. Unlike the time-integrated amplitude, the FT of the chirp shows a substantial contribution from higher frequency components ($>1\omega_{0}$).

To compare our results to a nonperturbative simulation of our experiment, we solve the SBE for MgO with our experimental parameters as inputs (see Materials and Methods and the Supplementary Materials for details) (*12*–*14*). Unlike perturbative models, the SBE framework can separate contributions from intra- and interband currents. The time-delay Fourier transforms of the real time-integrated amplitude and the temporal chirp, corresponding to the electric fields generated by intra- and interband currents, are shown in Fig. 3 (E and F). These plots show that the inter- and intraband contributions to the DFWM signal field observables are comparable under our experimental conditions.

The simple model of nonlinear susceptibility modulation discussed earlier provides physical insight into the emergence of peaks at integer multiples of $\omega_{0}$ observed in both the experimentally measured and theoretically calculated field observables in Fig. 3. The microscopic nonlinear susceptibility corresponding to the DFWM process $\chi^{\left(3\right)}\left(-\omega ;\omega ,-\omega ,\omega \right)\propto \frac{1}{{\left[\epsilon_{10}\left(q\right)-\hbar \omega -i\Gamma_{10}\right]}^{2}}$ depends on the energy gap between valence and conduction bands [$\epsilon_{10}\left(q\right)$], where $\Gamma_{10}$ is the coherence dephasing term. In the presence of the strong field, the band modulation leads to an instantaneous change in the energy gap $\epsilon_{10}\left[q+eA\left(t\right)\right]$, as has been explained earlier. This leads to modification of the response function, which becomes a functional of the total input electric field (see the Supplementary Materials for a detailed derivation). The total electric field E is the sum of the two Gate pulses ($E_{1}$ and $E_{2}$) and the Probe pulse ($E_{3}$) electric field. With an introduction of time delay (τ) between $E_{1}$ (phase locked with $E_{2}$) and $E_{3}$, the three pulses ${\tilde{E}}_{1},{\tilde{E}}_{2}$, and ${\tilde{E}}_{3}$ in the frequency domain can be written as

$$E_{1}\left(t-\tau \right)↔{\tilde{E}}_{1}\left(\omega \right)e^{i\left(k_{1}\cdot r-\omega \tau \right)}+c.c.$$ (4A)

$$E_{2}\left(t-\tau \right)↔{\tilde{E}}_{2}\left(\omega \right)e^{i\left(k_{2}\cdot r-\omega \tau \right)}+c.c.$$ (4B)

$$E_{3}\left(t\right)↔{\tilde{E}}_{3}\left(\omega \right)e^{ik_{3}\cdot r}+c.c.$$ (4C)

where $k_{i}$ is the wave vector of the ith field. It can be shown that the microscopic third-order nonlinear response depends on the total electric field resulting from band modulation as

$$\begin{array}{c} \chi^{\left(3\right)}\left(-\omega ;\omega ,-\omega ,\omega ;[\tilde{E}]\right)=\chi_{0}^{\left(3\right)}\left(-\omega ;\omega ,-\omega ,\omega \right)+\int \frac{d\Omega }{2\pi }a\left(\omega ,\Omega \right)\tilde{E}\left(\Omega \right)\\ +\iint \frac{d\Omega d\Omega^{\prime }}{{\left(2\pi \right)}^{2}}b\left(\omega ,\Omega ,\Omega^{\prime }\right)\tilde{E}\left(\Omega \right)\tilde{E}\left(\Omega^{\prime }\right)+O\left({\tilde{E}}^{3}\right) \end{array}$$ (5)

where $\chi_{0}^{\left(3\right)}$ is the field-free third-order susceptibility. The parameters *a* and *b* represent the coefficients governing the first- and second-order corrections of the nonlinear susceptibility. $\Omega \;\text{and}\;\Omega^{\prime }$ are integration variables. Plugging in all the relations, we get the expression of an effective DFWM polarization as

$$\begin{array}{c} P_{\text{eff}}^{\left(3\right)}=\{\chi_{0}^{\left(3\right)}+\int \frac{d\Omega }{2\pi }a\left(\omega ,\Omega \right)\left[{\tilde{E}}_{1}\left(\Omega \right)e^{-i\Omega \tau }+{\tilde{E}}_{2}\left(\Omega \right)e^{-i\Omega \tau }+{\tilde{E}}_{3}\left(\Omega \right)+c.c.\right]\\ +\iint \frac{d\Omega d\Omega^{\prime }}{{\left(2\pi \right)}^{2}}b\left(\omega ,\Omega ,\Omega^{\prime }\right)[{\tilde{E}}_{1}\left(\Omega \right)e^{-i\Omega \tau }+{\tilde{E}}_{2}\left(\Omega \right)e^{-i\Omega \tau }+{\tilde{E}}_{3}\left(\Omega \right)+c.c.]\times \\ \left[{\tilde{E}}_{1}\left(\Omega^{\prime }\right)e^{-i\Omega^{\prime }\tau }+{\tilde{E}}_{2}\left(\Omega^{\prime }\right)e^{-i\Omega^{\prime }\tau }+{\tilde{E}}_{3}\left(\Omega^{\prime }\right)+c.c.\right]\\ +\cdots \}{\tilde{E}}_{1}\left(\omega \right){\tilde{E}}_{2}^{*}\left(\omega \right){\tilde{E}}_{3}\left(\omega \right)e^{i\left(k_{1}-k_{2}+k_{3}\right)\cdot r} \end{array}$$ (6)

Here, the phase-matching conditions arising from terms that include the wave vectors $k_{i}$ corresponding to the input fields only apply to the wave-mixing terms outside the brackets. We do not include wave-vector terms in the field-dependent expansion of $\chi^{\left(3\right)}$ because the field-driven modification of $\chi^{\left(3\right)}$ is an instantaneous field effect independent of the direction of propagation of the field(s). This allows us to model nonperturbative field modification of the response function within perturbation theory and is distinct from a higher-order perturbation expansion shown in Eq. 3. Using this model, we can also derive an analytical expression for the temporal chirp (see the Supplementary Materials for a detailed derivation).

It can be readily seen from Eq. 6 that the $1\omega_{0}$ peak in Fig. 3 primarily originates from the linear expansion of the net field-dependent $\chi^{\left(3\right)}$. The second-order expansion contributes not only to the $1\omega_{0}$ peak but also generates a component at $2\omega_{0}$. A similar reasoning extends naturally to the higher-order expansions of $\chi^{\left(3\right)}$. Within this framework, the difference in the relative peak strengths at $1\omega_{0}$ and $2\omega_{0}$ can be understood by noting that the third-order susceptibility is predominantly governed by the linear term, i.e., $\chi^{\left(3\right)}\approx \chi_{0}^{\left(3\right)}+aE$. Analogous phenomena have previously been observed in which an external field modifies both the linear and nonlinear response functions of a material. A well-known example is the optical Kerr effect, in which the change in the linear refractive index of a medium is proportional to the intensity of the applied field (*48*–*50*). Similar effects have also been used in electric field–induced second-harmonic generation, where the external field breaks the inversion symmetry of otherwise centrosymmetric systems, making the second-order response function nonzero (*51*, *52*). In the framework of perturbation theory, a field-dependent nonlinear response is thus a good model to explain the observed oscillations in our experiment, whereas field-independent fifth-order and higher terms consistent with our phase-matching conditions are insufficient to explain the observations. Additional supporting evidence for our interpretation is presented in section S6. Furthermore, the field-dependent nonlinear response model explains the higher sensitivity of phase observables such as chirp to the strong-field driven modulation. The time-integrated amplitude is dominated by the large field-free contribution from $\chi_{0}^{\left(3\right)}$, with the field-dependent corrections ($a\cdot E,b\cdot E^{2},\cdots$) appearing as small modulations on top of this non-oscillatory background. As a result, only the leading $1\omega_{0}$ component is clearly visible in the Fourier transform of the integrated amplitude. The temporal chirp b(τ), in contrast, is extracted as a coefficient of a local polynomial fit to the temporal phase and does not sit on top of the same large field-free background because the input field chirp is small. The τ-dependent modulation of chirp is a direct readout of the field-dependent reshaping of the signal’s spectral phase.

### Subcycle control of dephasing

Ultrafast spectroscopies such as attosecond transient absorption and HHG have enabled real-time tracking of electronic coherences, providing direct access to dephasing dynamics that are otherwise obscured by inhomogeneous broadening in frequency-domain measurements. In solids, reported dephasing times span a substantial range, from a few hundred attoseconds in extreme ultraviolet transient absorption spectroscopy (*47*, *53*) to tens of femtoseconds in HHG and nonlinear optical measurements (*54*–*56*), and in the picosecond regime in field-resolved spectroscopy of liquids (*57*, *58*) and in emerging petahertz field-resolved spectroscopies of excitons (*31*). Theoretical descriptions have largely relied on the framework developed for disordered semiconductors (*59*, *60*), while measurements of the radiated electric field offer a more unambiguous route to the dephasing time, as demonstrated recently (*31*).

From perturbation theory, the third-order polarization can be written as (*16*, *61*)

$$P^{\left(3\right)}\left(t\right)=\text{Tr}\left[{\hat{\mu }\hat{\rho }}^{\left(3\right)}\left(t\right)e^{-t/\tau_{\text{dephase}}}\right]$$ (7)

where $\hat{\mu }$ is the transition dipole operator, $\rho^{\left(3\right)}\left(t\right)$ is the third-order density matrix in the nonlinear interaction, and $\tau_{\text{dephase}}$ is the dephasing time.

**Fig. 4.**
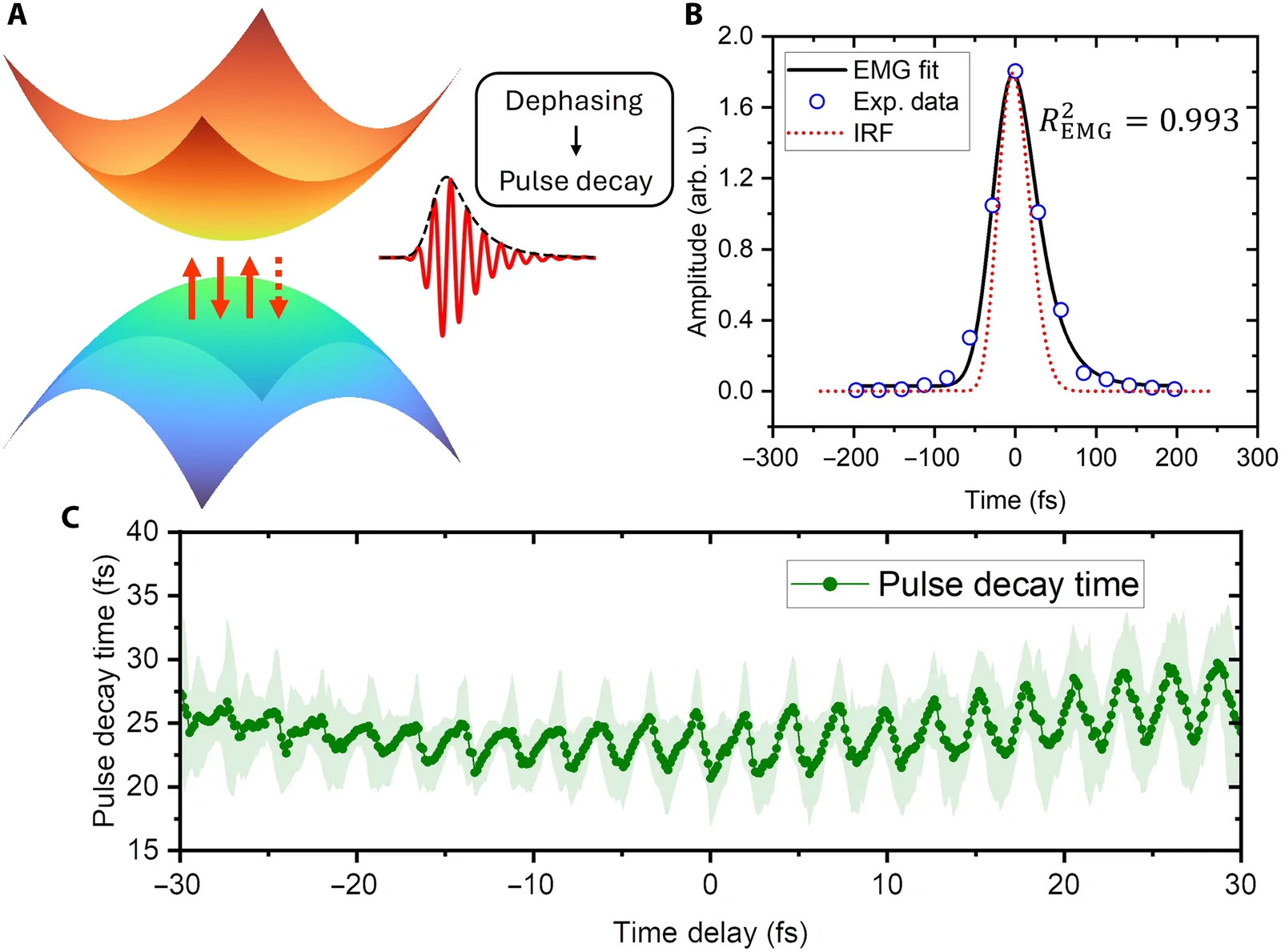
Extraction of dephasing times. (**A**) Schematic of DFWM in MgO, where the asymmetry in the output pulse contains the information of third-order coherence dephasing. The asymmetry shown in the output pulse is exaggerated for illustrative purposes. (**B**) A representative plot of the real-time amplitude of the measured output electric field at a time delay of 26 fs fitted with an EMG function (black, solid curve) is shown. The red, dotted curve corresponds to the IRF of the measurement. See the Supplementary Materials for details on the fitting procedure. (**C**) The pulse decay time, which corresponds to the dephasing time, as a function of time delay, is extracted, showing that strong light-matter interaction leads to attosecond modulation and control of dephasing. The shaded region indicates the standard error obtained by fitting the retrieved electric field across eight scans.

In the density matrix formalism, the diagonal terms correspond to the population of electronic states, and the off-diagonal elements represent coherence between states. As coherences evolve in time, phase fluctuations attenuate their temporal evolution because of dephasing (*61*). To model dephasing of coherences, phenomenological dephasing terms $\gamma_{\text{dephase}}=1/\tau_{\text{dephase}}$ are included in the nonlinear response. As seen from Eq. 7, the third-order polarization, which emits the nonlinear signal field, is related to the dephasing time. This means that the output-field pulse envelope width depends on the dephasing of the coherence created by the Probe pulse in the final step of the nonlinear interaction. To quantify third-order coherence dephasing, we fit the measured output temporal field with an exponentially modified Gaussian (EMG) function. We fix the full width at half maximum of the Gaussian function to 43 fs, corresponding to the full width at half maximum of the instrument response function (IRF) obtained by multiplying the temporal electric fields of the measured Gate and Probe pulses. We then extract the pulse decay time of the DFWM signal field as shown in Fig. 4B (see the Supplementary Materials for details on fitting). In a nonlinear interaction involving femtosecond pulses, multiple factors such as group-delay dispersion, phase matching bandwidth, spectrometer response, and imperfections in the input pulse shape could influence the shape of the emitted Signal pulse. In our experiment, pulse broadening due to group-delay dispersion in MgO is negligible (see the Supplementary Materials), and phase matching bandwidth spans the bandwidth of the input pulses. The home-built spectrometer is calibrated using a commercial spectrometer, and the measured input pulses influence the output via the IRF, which is shorter in duration than the measured signal electric field, as shown in Fig. 4B. The IRF is obtained by multiplying the measured electric field of the Gate pulses with that of the Probe (see the Supplementary Materials), corresponding to a third-order interaction.

The extracted pulse decay times corresponding to dephasing times $\tau_{\text{dephase}}$ from the EMG fit are plotted as a function of time delay in Fig. 4C. The shaded area represents the standard error obtained by fitting eight different time delay scans in the experiment. The data indicate that the subcycle delay in the strong light-matter interaction modulates the measured dephasing times of the third-order coherence. Multiple factors could influence dephasing times in solids, including doping, electron-electron interactions, and electron-phonon interactions. While the strong field–driven band structure modulation described in this study could modify these dephasing contributions, more detailed theoretical modeling of dephasing is necessary to establish such a connection (*62*). The work presented here demonstrates that dephasing times can be controlled on subcycle timescales in a nonlinear interaction where nonperturbative effects cannot be ignored.

In summary, we have measured the complete electric field of the DFWM signal in MgO using spectral interferometry. By analyzing field observables, such as the time-integrated amplitude and the signal field chirp, as functions of the subcycle time delay between incoming pulses, we identify unique oscillatory features. We attribute these features to the ultrafast modulation of the nonlinear response function of the crystal resulting from modulation of the band structure. To support this interpretation, we simulate the DFWM experiment using time-dependent perturbation theory. We find that oscillatory behavior is reproduced when instantaneous ultrafast band-structure modulation driven by the strong field is included. Such ultrafast band modulation effects are known to leave signatures in other optical observables, such as high-harmonic spectra (*34*, *35*, *63*). Here, we show that the influence of field-dressed bands is clearly manifested in the field observables of nonlinear optical signals. Our results indicate that field observables can serve as powerful probes of strong field–driven attosecond electron dynamics in solid-state systems. We have demonstrated that modeling of our experimental observations requires incorporating strong light-matter interactions into the nonperturbative modification of the third-order nonlinear susceptibility. Furthermore, by fitting the real-time electric field envelope of the Signal pulses, we have extracted dephasing times corresponding to this nonresonant nonlinear interaction. The modulation of the band structure is seen to modulate the dephasing time, resulting in subcycle control of dephasing. While this study demonstrates the high sensitivity of DFWM field observables to subcycle strong-field dressing, the detailed microscopic decomposition of all contributing pathways is identified as a promising direction for future work. The measurement of dephasing in a nonlinear process via electric field measurement could open doors to enhancing our understanding of ultrafast dephasing mechanisms in a wide variety of atomic, molecular, and material systems.

Our findings have important implications for the manipulation and control of observables governed by the nonlinear response function. For instance, nonlinear up-conversion of starlight has major benefits for astronomy (*64*) but is extremely challenging because of the low intensity of starlight. A strong field–enhanced nonlinear response could markedly improve up-conversion efficiency. In quantum optics, it is well established that signals generated through nonlinear optical processes can serve as sources of nonclassical light. Processes such as parametric down-conversion and DFWM are known to produce squeezed light (*65*–*69*). The degree of squeezing in these processes is directly proportional to the nonlinear coupling coefficients of the respective systems, i.e., $\propto \chi^{\left(2\right)}$ for parametric down-conversion and $\propto \chi^{\left(3\right)}$ for DFWM (*69*). It has been demonstrated that squeezing in DFWM signals can also be observed from ultrafast light-matter interactions (*70*–*72*). With the strong subcycle modulation of $\chi^{\left(3\right)}$ demonstrated in this work, it is now possible to control the degree of squeezing on attosecond timescales (*72*). This could pave the path to achieve extreme levels of squeezing on the basis of field-induced nonlinearities that exceed current squeezing levels achieved using periodically poled nonlinear materials (*73*) with numerous applications in quantum metrology, quantum spectroscopy, and quantum information science.

## MATERIALS AND METHODS

### Experimental setup

**Fig. 5.**
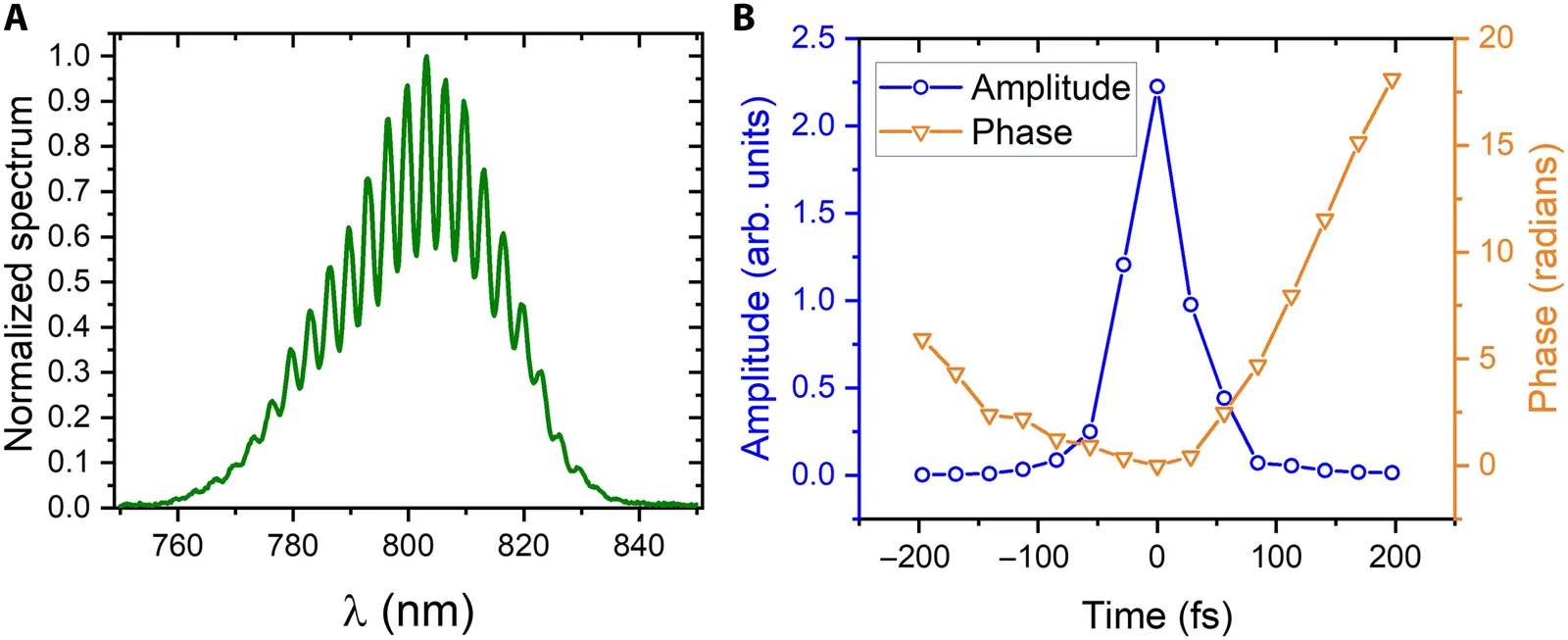
Extracted electric field using TADPOLE. (**A**) Representative plot of the experimental spectral interference data for the DFWM signal at a fixed time delay between Gate and Probe pulses. (**B**) Retrieved electric field temporal amplitude and phase from the spectral interference data in (A) using the measured amplitude and phase for the Reference pulse. The time resolution for the retrieved electric field is limited by the bandwidth of spectral interference and is 28 fs in our measurement. The Reference pulse is measured using the FROG technique, as shown in the Supplementary Materials (fig. S2A).

In our setup of the DFWM experiment (*27*), as seen in Fig. 1, three pulses with different propagation vectors interact with the MgO sample in a noncollinear geometry and generate a fourth pulse resulting from a third-order nonlinear interaction in a BOXCAR configuration (*32*). Because of the noncollinear geometry, the Signal pulse is spatially separated from the other three beams. The output signal has a momentum of $k_{s}=k_{1}-k_{2}+k_{3}$. The Probe and Gate beams are centered at 800 nm, with a pulse width of 50 fs and a repetition rate of 1 kHz. The Gate pulse is divided into two phase-locked Gate pulses (Gate 1 and Gate 2) using a mask. The Probe pulse is delayed with respect to the Gate pulses using an optical delay stage, and the Gate polarization can be rotated relative to the Probe polarization. However, in this experiment, the polarizations of all four pulses are fixed along the same direction. All the pulses interact with the sample with a relative angle of <1° and a focal spot size of ∼25 μm, resulting in a small time delay smearing of ∼600 as. The MgO crystal is mounted on a controllable rotation mount to allow precise control of the crystal angle relative to the Probe polarization. The Probe pulse and the two Gate pulses each have a pulse energy of ∼500 nJ and form the input pulses in our DFWM measurement. Before the interaction, a Reference pulse is derived from the Probe pulse. After the interaction, the Signal pulse is isolated by an iris, so the residual Probe and Gate beams are blocked, resulting in negligible scattered light measured with the Signal pulse. The Signal pulse passes through a high-contrast polarizer, which extracts the polarization component of the signal along the input polarization direction. The passive interferometric stability required for the subcycle time delay scanning in the experiment lasts for more than 72 hours in our setup. The output Signal pulse is completely measured using TADPOLE (*29*), where the Reference pulse is combined with the Signal pulse in a spectrometer to obtain the measurement of spectral interference (*27*, *28*), as shown in Fig. 5A. Frequency-resolved optical gating (FROG) (*74*) is used to completely characterize the Reference pulse (see fig. S2A). The measured spectral interference of the DFWM signal and the Reference pulse can be written as

$$S\left(\omega \right)=S_{R}\left(\omega \right)+S_{S}\left(\omega \right)+\sqrt{S_{R}\left(\omega \right)}\sqrt{S_{S}\left(\omega \right)}\text{cos}\left[\varphi_{S}\left(\omega \right)-\varphi_{R}\left(\omega \right)+\omega \;\tau_{R}\right]$$ (8)

S(ω) is the spectral intensity, ω is the angular frequency, and φ(ω) is the spectral phase. The subscripts S and R stand for signal and reference, respectively. $\tau_{R}$ is the time delay between the Reference and Signal pulses. From the measured spectral interference data (Fig. 5A) and using the measured Reference pulse spectral amplitude and phase, $S_{S}\left(\omega \right)$ and $\phi_{S}\left(\omega \right)$ are retrieved by performing a Fourier transform, followed by filtering and phase extraction (*27*). The frequency domain field amplitude and phase are used to obtain the time domain field by performing a Fourier transform. This provides the temporal amplitude and phase of the signal field, as shown in Fig. 5B. The time resolution of the retrieved temporal electric field is limited by the bandwidth of the spectral fringes and is 28 fs in our experiment. While the FROG technique offers a higher time resolution in electric field measurement, the low signal levels (∼1 nJ) in our experiment make FROG unsuitable. Although petahertz field sampling techniques are capable of measuring approximately femtojoule-level pulses (*75*), they typically do not work in a single-shot configuration and require time delay scanning for measuring the field, making them unsuitable for our experiment.

### Simulation

#### Time-dependent perturbation theory

To simulate the complete electric field of the DFWM signal, we use time-dependent perturbation theory to evaluate the third-order expectation value of the microscopic current operator. The streaking of the electron momentum, $p\left(t\right)=p_{0}+eA_{\text{ext}}\left(t\right)$, is explicitly incorporated. The emitted DFWM signal is expressed as $E_{\text{DFWM}}\left(t\right)\propto \frac{\partial }{\partial t}{\langle J\left(t\right)\rangle }^{\left(3\right)}$, where

$$\begin{array}{c} {\langle J^{i}\left(t\right)\rangle }^{\left(3\right)}=\sum_{\alpha ,\beta ,\gamma ,q\in \text{BZ}}N_{q}^{i\alpha \beta \gamma }\int_{t_{0}}^{t}{\mathrm{dt}}_{3}\int_{t_{0}}^{t_{3}}{\mathrm{dt}}_{2}\int_{t_{0}}^{t_{2}}{\mathrm{dt}}_{1}\;A_{\text{ext}}^{\alpha }\left(t_{1}\right)A_{\text{ext}}^{\beta }\left(t_{2}\right)A_{\text{ext}}^{\gamma }\left(t_{3}\right)\\ \times \text{exp}\left[i\int_{t_{0}}^{t}\Theta_{0,1,q}\left(t^{\prime }\right)dt^{\prime }+i\int_{t_{0}}^{t_{1}}\Theta_{1,0,q}\left(t^{\prime }\right)dt^{\prime }+i\int_{t_{0}}^{t_{2}}\Theta_{0,1,q}\left(t^{\prime }\right)dt^{\prime }+i\int_{t_{0}}^{t_{3}}\Theta_{1,0,q}\left(t^{\prime }\right)dt^{\prime }\right] \end{array}$$ (9)

Here, $\Theta_{1,0,q}\left(t\right)$ is the time-dependent energy gap, defined as $\Theta_{1,0,q}\left(t\right)=\epsilon_{\text{CB}}\left(t\right)-\epsilon_{\text{VB}}\left(t\right)$. Here, 0 and 1 denote the valence band (VB) and conduction band (CB), respectively, in the two-band model, and i,α,β, and γ index the polarization directions. Further details of the derivation are provided in the Supplementary Materials.

#### Semiconductor Bloch equation

We use a microscopic model on the basis of SBE to numerically simulate the DFWM experiment, explicitly accounting for the nonperturbative effects of the strong field on the material (*12*–*14*). The SBE is formulated in the length gauge (*45*) for a two-band system consisting of the highest valence band and the lowest conduction band of MgO, as obtained from a one-dimensional tight-binding model (*9*). The restriction to two bands is justified by the off-resonant driving conditions ($\hbar \omega \ll \Delta_{\text{gap}}$), which strongly suppress excitation pathways involving higher conduction bands.

The transient electron dynamics under a strong, time-dependent electric field are described using the SBE, which governs the evolution of the electron and hole populations, $n_{k}^{e}$ and $n_{k}^{h}$, as well as the microscopic polarization $p_{k}^{h,e}$ between them (*12*–*14*)

$$\hbar \frac{\partial n_{k}^{e}}{\partial t}=-2\hbar ℑ\left[d_{k}^{\mathrm{cv}}E\left(t\right)\right]+|e|E\left(t\right)\nabla_{k}n_{k}^{e}-\frac{1}{2T_{1}}\left(n_{k}^{e}-n_{-k}^{e}\right)$$ (10A)

$$\hbar \frac{\partial n_{k}^{h}}{\partial t}=-2\hbar ℑ\left[d_{k}^{\mathrm{cv}*}E\left(t\right)\right]+|e|E\left(t\right)\nabla_{k}n_{k}^{h}-\frac{1}{2T_{1}}\left(n_{k}^{h}-n_{-k}^{h}\right)$$ (10B)

$$\begin{array}{c} i\hbar \frac{\partial p_{k}^{h,e}}{\partial t}=\left(\epsilon_{k}^{e}+\epsilon_{k}^{h}-i\frac{\hbar }{T_{2}}\right)p_{k}^{h,e}-d_{k}^{\mathrm{cv}}E\left(t\right)\left(1-n_{k}^{e}-n_{k}^{h}\right)\\ +i|e|E\left(t\right)\nabla_{k}p_{k}^{h,e} \end{array}$$ (10C)

Here, $d_{k}^{\mathrm{cv}}$ denotes the transition dipole matrix element between the conduction and valence bands. The quantities $\epsilon_{k}^{e}$ and $\epsilon_{k}^{h}$ represent the electron and hole dispersion relations, defined as $\epsilon_{k}^{e}=\epsilon_{k}^{c}$ and $\epsilon_{k}^{h}=-\epsilon_{k}^{v}$, where $\epsilon_{k}^{c}$ and $\epsilon_{k}^{v}$ are the conduction and valence band energies, respectively. The phenomenological relaxation and dephasing times are set to $T_{1}=7\;\text{fs}$ and $T_{2}=1.1\;\text{fs}$ (*13*, *14*). The pulse decay time measured in our experiment corresponds to the third-order coherence dephasing and is distinct from the $T_{2}$ time used in the SBE calculations. The calculation includes only coherent single-particle dynamics and neglects excitonic correlations, carrier-carrier scattering, and other many-body renormalizations. The decay and dephasing times are incorporated phenomenologically. Propagation effects are not included; however, because of the thin-sample geometry and moderate intensities used, these effects are not expected to appreciably influence the main features of the calculated response.

The emitted field is proportional to the current source $J_{\text{intra}}\left(t\right)=\frac{e}{\hbar }\sum_{k,i}\nabla_{k}\epsilon_{k}^{i}n_{k}^{i}$ and to a polarization source $P_{\text{inter}}\left(t\right)=\sum_{k}d_{k}^{\mathrm{cv}}p_{k}^{h,e}$, corresponding to the intra- and interband currents, respectively, as shown in Fig. 3 (E and F). The calculation reproduces the subcycle oscillations observed in the field observables. Further implementation details are provided in the Supplementary Materials.

## Supplementary Materials

### This PDF file includes:

Supplementary Text Figs. S1 to S8 References
