# Supplementary material for: Strong field–driven subcycle band structure modulation and dephasing control
Source: Sci Adv. 2026 Aug 28;12(35):eaeg3966. doi: 10.1126/sciadv.aeg3966 (PMC13524020; doi:10.1126/sciadv.aeg3966)
Supplement: Supplementary file 1 [file sciadv.aeg3966_sm.pdf]

Science Advances
AAAS

Supplementary Materials for

Strong field–driven subcycle band structure modulation and dephasing control

Francis Walz *et al.*

Corresponding author: Shashank Kumar, kumar414@purdue.edu; Niranjan Shivaram, niranjan@purdue.edu

Sci. Adv. **12**, eaeg3966 (2026)
DOI: 10.1126/sciadv.aeg3966

This PDF file includes:

Supplementary Text
Figs. S1 to S8
References

1 Exponentially modified Gaussian fitting

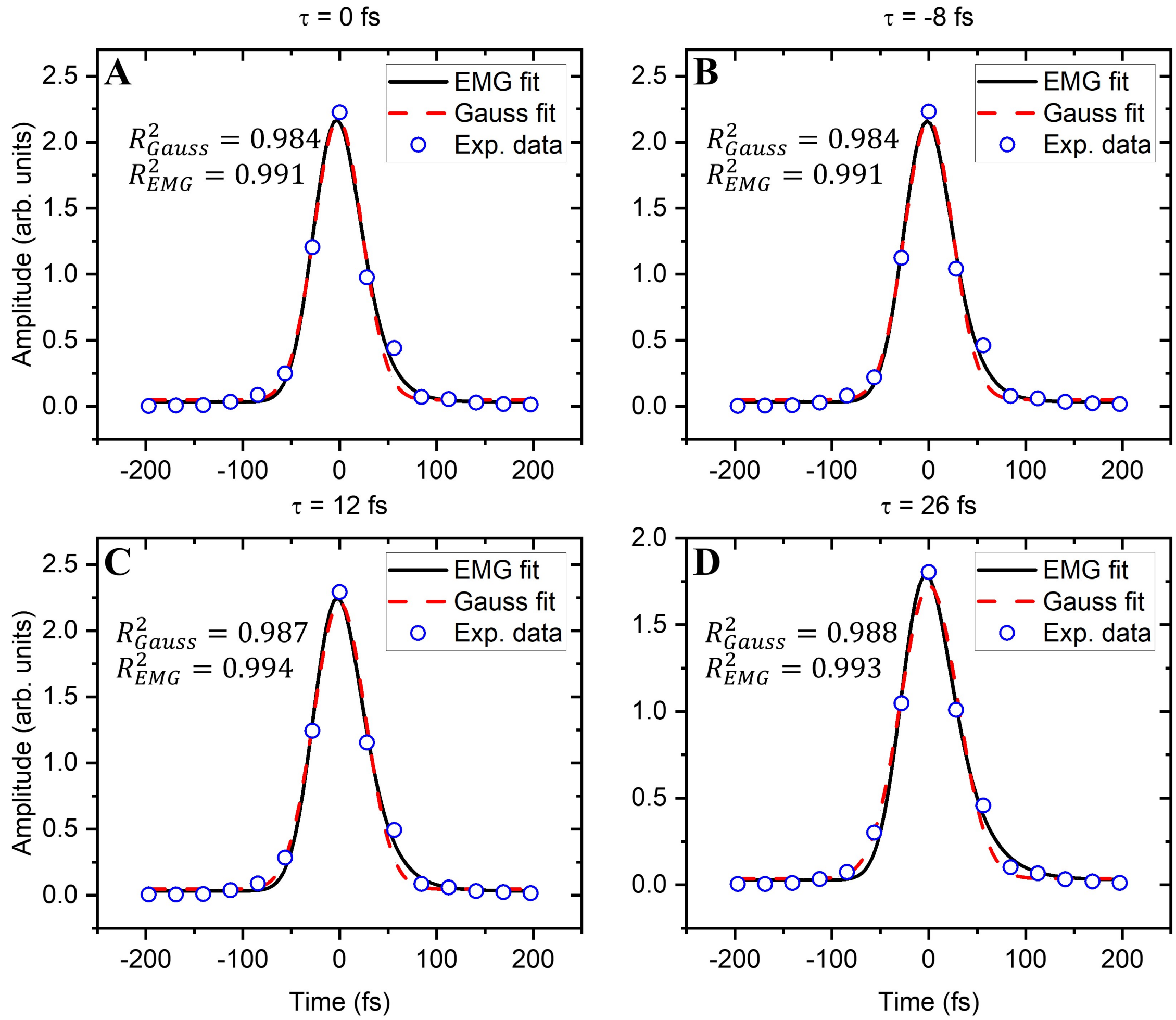

Figure S1: **Comparison of EMG and Gaussian fit**: Fitting of temporal amplitude of DFWM signal at (A) $\tau = 0$ fs, (B) $\tau = -8$ fs, (C) $\tau = 12$ fs, and (D) $\tau = 26$ fs using Gaussian (red dashed curve) and Exponentially modified Gaussian (black solid curve) functions. The goodness of both fits is also shown in each plot.

In Fig. 2 A, the DFWM signal amplitude has an approximately Gaussian form in real-time for each time delay. However, a small asymmetry is observed around zero time (see Fig. S1) due to dephasing of the coherence generated in the final step of the DFWM process. Consequently, for each time delay, we fit both a simple Gaussian profile and an exponentially modified Gaussian

(EMG) function. The fitting functions are defined as,

$$f_{\text{Gauss}} = A \exp\left(-\frac{1}{2}\left(\frac{t-t_0}{\sigma}\right)^2\right) + C \tag{S1a}$$

$$f_{\text{EMG}} = A \exp\left(-\frac{1}{2}\left(\frac{t-t_0}{\sigma}\right)^2\right)\frac{\sigma}{\tau_{\text{dephase}}}\sqrt{\frac{\pi}{2}}\text{erfcx}\left(\frac{1}{\sqrt{2}}\left(\frac{\sigma}{\tau_{\text{dephase}}} - \frac{t-t_0}{\sigma}\right)\right) + C \tag{S1b}$$

where σ is the Gaussian width and τ_{dephase} corresponds to the dephasing time of the interaction. The scaled complementary error function is defined as $\text{erfcx}(t) = \exp(t^2)\,\text{erfc}(t)$, with $\text{erfc}(t)$ being the complementary error function. Fig. S1 shows the Gaussian (red dashed curve) and EMG (black solid curves) fitting along with the experimental data (blue markers) with a temporal resolution of 28 fs for a few different time delays. For the EMG fit, we fix the FWHM of 43 fs, which corresponds to the FWHM of the instrument response function (IRF) as shown in Fig. S2. The parameters are extracted from the fits and plotted as a function of time delay in Fig. S3. The EMG fit shows a higher R^2 value and is thus a better model for the signal pulse shape.

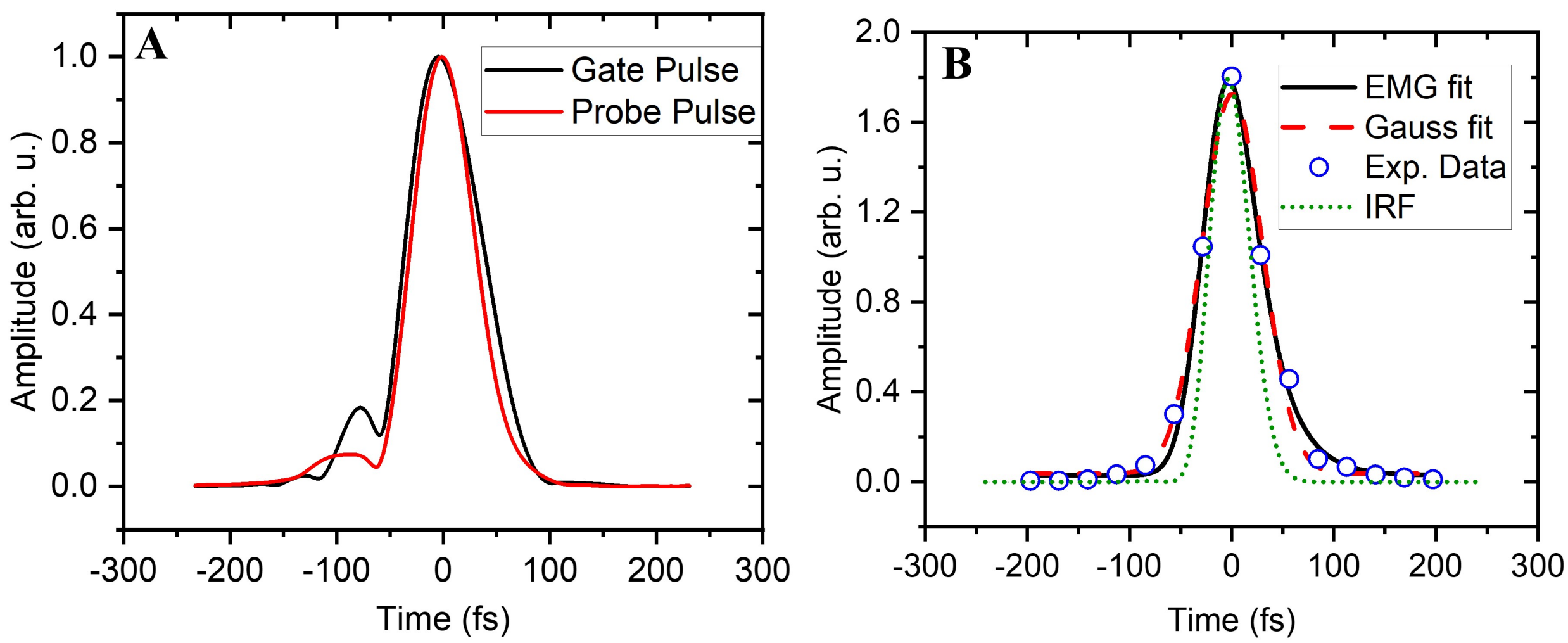

Figure S2: **Instrument response function from input fields**: (A) Input electric fields of the gate and probe pulses measured using FROG. (B) EMG fit with a fixed Gaussian width (FWHM) of 43 fs, corresponding to the instrument response function (IRF). The IRF is calculated as the product of E_{gate}^2 and E_{probe}.

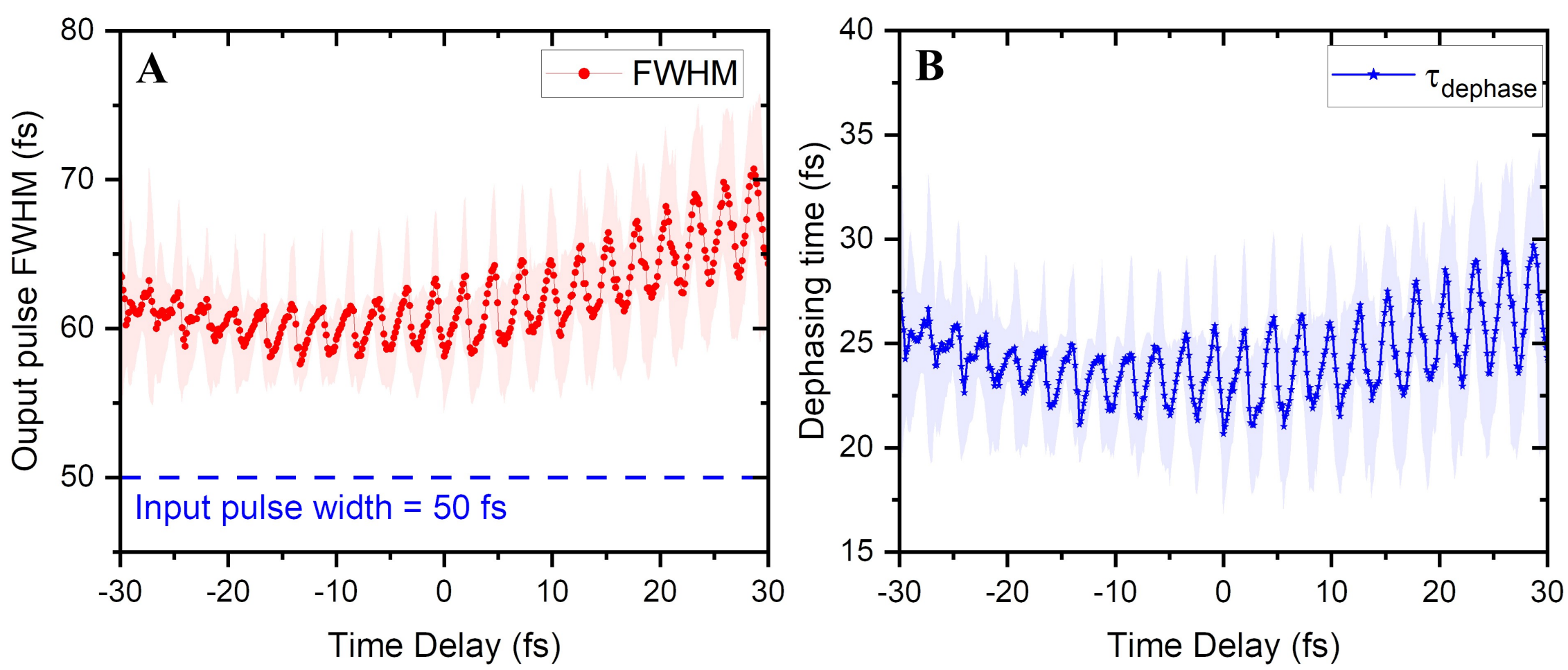

Figure S3: **Modulation of fitted parameters**: (A) Extracted output pulse width from the Gaussian fit as a function of time delay. (B) Extracted dephasing times τ_{dephase} (pulse decay times) from an Exponentially Modified Gaussian fit with a fixed Gaussian width (FWHM) of 43 fs corresponding to the IRF width. The shaded area represents the statistical error obtained by fitting the retrieved electric field across eight scans.

2 Nonlinear susceptibility modulation

The induced polarization spectrum $P(\omega)$ can be expressed as a power series of the electric field as,

$$P(\omega) = \epsilon_0[\chi^{(1)}\mathcal{E}(\omega) + \chi^{(3)}(\omega;\omega_1,\omega_2,\omega_3)\mathcal{E}(\omega_1)\mathcal{E}(\omega_2)\mathcal{E}(\omega_3) + \cdots] \tag{S2}$$

where $\chi^{(n)}$ is the nth order non-linear susceptibility tensor. The $\mathcal{E}(\omega)$ is the spectrum of the total incoming electric field. Here, we have excluded the even-order terms because they vanish for centrosymmetric materials. For the signal corresponding to a four-wave mixing process, like DFWM, we are interested in the terms $n \geq 3$. With an introduction of time delay (τ) between E_1 (phase-locked with E_2) and E_3, the three pulse E_1, E_2 and E_3 in frequency domain can be written as,

$$E_1(t-\tau) \leftrightarrow E_1(\omega)e^{i(\boldsymbol{k}_1\cdot\boldsymbol{r}-\omega\tau)} + c.c \tag{S3a}$$

$$E_2(t-\tau) \leftrightarrow E_2(\omega)e^{i(\boldsymbol{k}_2\cdot\boldsymbol{r}-\omega\tau)} + c.c \tag{S3b}$$

$$E_3(t) \leftrightarrow E_3(\omega)e^{i\boldsymbol{k}_3\cdot\boldsymbol{r}} + c.c \tag{S3c}$$

where $\boldsymbol{k}_i$ is the wave vector of the ith field. Plugging in the relation in Eq. S2 and selecting appropriate terms corresponding to the DFWM signal,

$$\begin{aligned} P_{DFWM} =& \chi_0^{(3)}(-\omega;\omega,-\omega,\omega)E_1(\omega)E_2^*(\omega)E_3(\omega)e^{i(\boldsymbol{k}_1-\boldsymbol{k}_2+\boldsymbol{k}_3)\cdot\boldsymbol{r}} \\ &+ \chi_0^{(5)}(|E_1|^2+|E_2|^2+|E_3|^2)E_1(\omega)E_2^*(\omega)E_3(\omega)e^{i(\boldsymbol{k}_1-\boldsymbol{k}_2+\boldsymbol{k}_3)\cdot\boldsymbol{r}} + \cdots \end{aligned} \tag{S4}$$

By substituting this expression into the wave equation, it can be shown that the DFWM signal is generated when the phase matching condition $\boldsymbol{k}_s = \boldsymbol{k}_1 - \boldsymbol{k}_2 + \boldsymbol{k}_3$ is satisfied as the three waves propagate through a finite-size nonlinear medium (*16*). However, the polarization doesn't exhibit any explicit $e^{-i\omega\tau}$ dependence, indicating that higher-order ($\chi_0^{(5)}$) wave mixing alone cannot account for the oscillatory behavior observed in the experiment.

To demonstrate the effects of strong-field interactions on the nonlinear susceptibility of the material, the microscopic $\chi_0^{(3)}(-\omega;\omega,-\omega,\omega)$ for the DFWM signal in MgO is derived using third-order polarization via perturbation theory (*61, 76, 16*).

$$\begin{aligned} P^{(3)}(t) = \sum_{\boldsymbol{q}}\int_0^\infty d\tau_3\int_0^\infty d\tau_2\int_0^\infty d\tau_1\ \mu_{01}(\boldsymbol{q})\mu_{10}(\boldsymbol{q})\mu_{01}(\boldsymbol{q})\mu_{10}(\boldsymbol{q}) \\ \times G_{10}(\tau_3,\boldsymbol{q})G_{00}(\tau_2)G_{10}(\tau_1,\boldsymbol{q})E_3(t-\tau_3)E_2(t-\tau_3-\tau_2)E_1(t-\tau_3-\tau_2-\tau_1) \end{aligned} \tag{S5}$$

Here, $\mu_{10}(\boldsymbol{q}) = \langle 1, \boldsymbol{q}|r|0, \boldsymbol{q}\rangle$ represents the transition dipole moment between the valence band ($|0, \boldsymbol{q}\rangle$) and the conduction band ($|1, \boldsymbol{q}\rangle$). $G_{10}(\tau_3, \boldsymbol{q})$ is the time propagator for coherence between the conduction and valence bands, while $G_{00}(\tau_3)$ is the population propagator. These propagators are defined as follows:

$$G_{10}(t, \boldsymbol{q}) = \Theta(t)e^{-i\epsilon_{10}(q)t-\Gamma_{10}t} \tag{S6a}$$

$$G_{00}(\tau_3) = \Theta(t)e^{-\Gamma_{00}t} \tag{S6b}$$

$\epsilon_{10}(\boldsymbol{q}) = \epsilon_{CB} - \epsilon_{VB}$ denotes the energy difference between the valence and conduction bands. Γ_{10} and Γ_{00} are phenomenological decay terms. The Fourier transformation of the third-order polarization yields:

$$\tilde{P}^{(3)}(\omega_s) = \sum_{\boldsymbol{q}} \mathcal{K}(\boldsymbol{q}) \int d\omega_3 \int d\omega_2 \int d\omega_1 \frac{1}{(2\pi)^3} \tilde{G}_{10}(\omega_1 + \omega_2 + \omega_3, \boldsymbol{q}) \tilde{G}_{00}(\omega_1 + \omega_2) \tilde{G}_{10}(\omega_1, \boldsymbol{q})$$
$$\times 2\pi\delta(\omega_s - \omega_1 - \omega_2 - \omega_3)\tilde{E}_1(\omega_1)\tilde{E}_2(\omega_2)\tilde{E}_3(\omega_3) \tag{S7}$$

$\mathcal{K}(q) = \mu_{01}(\boldsymbol{q})\mu_{10}(\boldsymbol{q})\mu_{01}(\boldsymbol{q})\mu_{10}(\boldsymbol{q})$ is introduced for brevity. $\tilde{G}_{10}$ and $\tilde{G}_{00}$ denote the Fourier transforms of the coherence and population propagators, respectively. This formulation indicates that the third-order response function $\chi^{(3)}$ is the product of three propagators:

$$\chi^{(3)}(-\omega_s; \omega_1, \omega_2, \omega_3) = \sum_{\boldsymbol{q}} \mathcal{K}(\boldsymbol{q}) \tilde{G}_{10}(\omega_1 + \omega_2 + \omega_3, \boldsymbol{q}) \tilde{G}_{00}(\omega_1 + \omega_2) \tilde{G}_{10}(\omega_1, \boldsymbol{q}) \tag{S8}$$

For DFWM signal, $\omega_1 = -\omega_2 = \omega_3 = \omega$, therefore the susceptibility corresponding to the DFWM process is,

$$\chi^{(3)}(-\omega; \omega, -\omega, \omega) = \sum_{\boldsymbol{q}} \mathcal{K}(\boldsymbol{q}) \tilde{G}_{10}(\omega, \boldsymbol{q}) \tilde{G}_{00}(0) \tilde{G}_{10}(\omega, \boldsymbol{q}) \tag{S9}$$

The Bloch-acceleration theorem states that electrons in the bands experience acceleration in the presence of the total external field, such that $\boldsymbol{q}(t) = \boldsymbol{q} + e\boldsymbol{A}_{ext}(t)$, where $\boldsymbol{A}_{ext}(t) = -\int dt\ \boldsymbol{\mathcal{E}}(t)$ is the total vector potential (with $\hbar = 1$). In the velocity gauge, this leads to field-dressed band effects, resulting in the modification of the band energy as $\epsilon_i(\boldsymbol{q}(t)) = \epsilon_i(\boldsymbol{q} + e\boldsymbol{A}_{ext}(t))$. Consequently, the coherence propagator can be expressed as a functional of the total vector potential of the external

field $\boldsymbol{A}$:

$$G_{10}(t,\boldsymbol{q};[\boldsymbol{A}]) = G_{10}^{(0)}(t,\boldsymbol{q})e^{-i\delta\Phi(t,\boldsymbol{q};[\boldsymbol{A}])} \tag{S10}$$

$$\text{where, } \delta\Phi = \int_{-\infty}^{t} dt'\ \epsilon_{10}(\boldsymbol{q}+e\boldsymbol{A}(t'))\ - \epsilon_{10}(\boldsymbol{q})t \tag{S11}$$

Here, $G_{10}^{(0)}(t,\boldsymbol{q})$ denotes the unperturbed propagator as defined in Eq. S6a. The phase deviation $\delta\Phi$ can be expanded as a power series in $\boldsymbol{A}$:

$$\delta\Phi(t,\boldsymbol{q};[\boldsymbol{A}]) \approx ev_\alpha(\boldsymbol{q})\int_{-\infty}^{t} dt'\ A_\alpha(t') + \frac{e^2}{2}m_{\alpha\beta}^{-1}(\boldsymbol{q})\int_{-\infty}^{t} dt'\ A_\alpha(t')A_\beta(t') + \cdots \tag{S12}$$

In this context, $v_\alpha(\boldsymbol{q}) = \frac{\partial\epsilon_{10}(\boldsymbol{q})}{\partial q_\alpha}$ is the band velocity, and $m_{\alpha\beta}^{-1}(\boldsymbol{q}) = \frac{\partial^2\epsilon_{10}(\boldsymbol{q})}{\partial q_\alpha \partial q_\beta}$ is the inverse-mass tensor. Summations over α, β, and similar indices are omitted for brevity. Equation S10 can be further expanded as a power series in $\delta\Phi$:

$$G_{10}(t,\boldsymbol{q};[\boldsymbol{A}]) = G_{10}^{(0)}(t,\boldsymbol{q})\left(1 - i\delta\Phi - \frac{1}{2}(\delta\Phi)^2 + \cdots\right) \tag{S13}$$

$$= G_{10}^{(0)}(t,\boldsymbol{q}) + G_{10}^{(1)}(t,\boldsymbol{q};[\boldsymbol{A}]) + G_{10}^{(2)}(t,\boldsymbol{q};[\boldsymbol{A}]) + \cdots \tag{S14}$$

From Eq. S12 and S13, the first-order correction (of the order of $\boldsymbol{A}$) to the modified coherence propagator is given by:

$$\begin{aligned} G_{10}^{(1)}(t,\boldsymbol{q};[\boldsymbol{A}]) &= -iG_{10}^{(0)}(t,\boldsymbol{q}) \times ev_\alpha(\boldsymbol{q})\int_{-\infty}^{t} dt'\ A_\alpha(t') \\ &= -iev_\alpha(\boldsymbol{q})G_{10}^{(0)}(t,\boldsymbol{q})\int_{-\infty}^{t} dt'\ \lim_{\eta\to 0}\int \frac{d\Omega}{2\pi}\tilde{A}_\alpha(\Omega)e^{-i\Omega t'+\eta t'} \\ &= ev_\alpha(\boldsymbol{q})G_{10}^{(0)}(t,\boldsymbol{q})\int \frac{d\Omega}{2\pi}\frac{\tilde{A}_\alpha(\Omega)}{\Omega}e^{-i\Omega t} \end{aligned} \tag{S15}$$

Accordingly, the Fourier transform of the first-order correction to the modified propagator is:

$$\tilde{G}_{10}^{(1)}(\omega,\boldsymbol{q};[\boldsymbol{A}]) = ev_\alpha(\boldsymbol{q})\int \frac{d\Omega}{2\pi}\frac{\tilde{G}_{10}^{(0)}(\omega-\Omega,\boldsymbol{q})}{\Omega}\tilde{A}_\alpha(\Omega) \tag{S16}$$

A similar expression can also be derived for second-order correction $G_{10}^{(2)}(\omega,\boldsymbol{q};[\boldsymbol{A}])$.

Assuming that the transition dipole matrix elements do not change with the band modification (The $\mathcal{K}(\boldsymbol{q})$ will not be the functional of $\boldsymbol{A}$), the third-order susceptibility $\chi^{(3)}$ from Eq. S9 is also

modified as follows:

$$\chi^{(3)}(-\omega;\omega,-\omega,\omega;[\boldsymbol{A}]) = \sum_{\boldsymbol{q}} \mathcal{K}(\boldsymbol{q})\tilde{G}_{10}(\omega,\boldsymbol{q};[\boldsymbol{A}])\tilde{G}_{00}(0)\tilde{G}_{10}(\omega,\boldsymbol{q};[\boldsymbol{A}]) \tag{S17}$$

$$\approx \sum_{\boldsymbol{q}} \mathcal{K}(\boldsymbol{q})\left(\tilde{G}^{(0)}_{10}(\omega,\boldsymbol{q}) + \tilde{G}^{(1)}_{10}(\omega,\boldsymbol{q};[\boldsymbol{A}]) + \tilde{G}^{(2)}_{10}(\omega,\boldsymbol{q};[\boldsymbol{A}]) + \cdots\right)$$

$$\times \tilde{G}_{00}(0)\left(\tilde{G}^{(0)}_{10}(\omega,\boldsymbol{q}) + \tilde{G}^{(1)}_{10}(\omega,\boldsymbol{q};[\boldsymbol{A}]) + \tilde{G}^{(2)}_{10}(\omega,\boldsymbol{q};[\boldsymbol{A}]) + \cdots\right) \tag{S18}$$

$$= \sum_{\boldsymbol{q}} \mathcal{K}(q)\left(\tilde{G}^{(0)}_{10}(\omega,\boldsymbol{q})\tilde{G}_{00}(0)\tilde{G}^{(0)}_{10}(\omega,\boldsymbol{q}) + 2\tilde{G}^{(1)}_{10}(\omega,\boldsymbol{q};[\boldsymbol{A}])\tilde{G}_{00}(0)\tilde{G}^{(0)}_{10}(\omega,\boldsymbol{q})\right.$$

$$\left. +2\tilde{G}^{(2)}_{10}(\omega,\boldsymbol{q};[\boldsymbol{A}])\tilde{G}_{00}(0)\tilde{G}^{(0)}_{10}(\omega,\boldsymbol{q}) + \tilde{G}^{(1)}_{10}(\omega,\boldsymbol{q};[\boldsymbol{A}])\tilde{G}_{00}(0)\tilde{G}^{(1)}_{10}(\omega,\boldsymbol{q};[\boldsymbol{A}]) + \cdots\right) \tag{S19}$$

$$= \chi^{(3)}_0(-\omega;\omega,-\omega,\omega) + \chi^{(3)}_1(-\omega;\omega,-\omega,\omega;[\boldsymbol{A}]) + \chi^{(3)}_2(-\omega;\omega,-\omega,\omega;[\boldsymbol{A}]) + \cdots \tag{S20}$$

Using the expression in Eq. S16, the first-order correction to the susceptibility is,

$$\chi^{(3)}_1(-\omega;\omega,-\omega,\omega;[\boldsymbol{A}]) = \sum_{\boldsymbol{q}} 2ev_\alpha(\boldsymbol{q})\mathcal{K}(\boldsymbol{q})\int \frac{d\Omega}{2\pi}\frac{1}{\Omega}\tilde{G}^{(0)}_{10}(\omega-\Omega,\boldsymbol{q})\tilde{G}_{00}(0)\tilde{G}^{(0)}_{10}(\omega,\boldsymbol{q})\tilde{A}_\alpha(\Omega) \tag{S21}$$

$$\chi^{(3)}_1(-\omega;\omega,-\omega,\omega;[\boldsymbol{\mathcal{E}}]) = \sum_{\boldsymbol{q}} 2ev_\alpha(\boldsymbol{q})\mathcal{K}(\boldsymbol{q})\int \frac{d\Omega}{2\pi}\frac{1}{i\Omega^2}\tilde{G}^{(0)}_{10}(\omega-\Omega,\boldsymbol{q})\tilde{G}_{00}(0)\tilde{G}^{(0)}_{10}(\omega,\boldsymbol{q})\tilde{\mathcal{E}}_\alpha(\Omega) \tag{S22}$$

$$= \int \frac{d\Omega}{2\pi} a_\alpha(\omega,\Omega)\mathcal{E}_\alpha(\Omega) \tag{S23}$$

In the final step, $\tilde{A}(\omega) = \frac{1}{i\omega}\tilde{\mathcal{E}}(\omega)$ is used to show that the modified susceptibility can be expressed equivalently as a functional of the total electric field. Similarly, the expression for the second-order correction to the susceptibility can be derived:

$$\chi^{(3)}_2(-\omega;\omega,-\omega,\omega;[\boldsymbol{\mathcal{E}}]) = \iint \frac{d\Omega d\Omega'}{(2\pi)^2} b_{\alpha\beta}(\omega,\Omega,\Omega')\mathcal{E}_\alpha(\Omega)\mathcal{E}_\beta(\Omega') \tag{S24}$$

Assuming $a(\omega,\Omega) \approx \delta(\omega-\Omega)$ and $b(\omega,\Omega,\Omega') \approx \delta(\omega-\Omega)\delta(\omega-\Omega')$, the nonlinear susceptibility can be further simplified $\chi^{(3)}(-\omega;\omega,-\omega,\omega) \approx \chi^{(3)}_0 + a\tilde{\mathcal{E}} + b\tilde{\mathcal{E}}^2 + \cdots$, where the α indices has been dropped as the input electric fields have same polarization. This approximation says that the dressing field's effect on $\chi^{(3)}$ at probe frequency ω is dominated by the field's spectral component at the probe frequency itself. Therefore, the modified DFWM polarization becomes,

$$P^{(3)}_{eff} = \left[\chi^{(3)}_0 + a\left(\tilde{E}_1(\omega)e^{-i\omega\tau} + \tilde{E}_2(\omega)e^{-i\omega\tau} + \tilde{E}_3(\omega) + c.c.\right) + \cdots\right]\tilde{E}_1(\omega)\tilde{E}^*_2(\omega)\tilde{E}_3(\omega) \tag{S25}$$

where $\chi_0^{(3)}$ is the unperturbed susceptibility. Expanding this relation gives,

$$P^{(3)}_{eff} = f_0(\omega) + f_1(\omega)e^{-i\omega\tau} + g_1(\omega)e^{i\omega\tau} + f_2(\omega)e^{-2i\omega\tau} + g_2(\omega)e^{2i\omega\tau} + \cdots \tag{S26}$$

Upon inverse transformation, the time-dependent third-order polarization will have terms that depend on $\cos\omega\tau$ due to the phase $e^{-i\omega\tau}$ in the spectrum. This means that the amplitude of the DFWM output signal will oscillate as a function of the time delay, as is evident in the experiment.

Now similarly we can calculate the spectral phase of the third-order polarization as,

$$\phi(\omega) = \arctan\left(\frac{\Im(P^{(3)})}{\Re(P^{(3)})}\right) \tag{S27}$$

$$= \arctan\left(\frac{\substack{\Im(f_0)+(\Im(f_1)+\Im(g_1))\cos\omega\tau-(\Re(f_1)-\Re(g_1))\sin\omega\tau \\ +(\Im(f_2)+\Im(g_2))\cos 2\omega\tau-(\Re(f_2)-\Re(g_2))\sin 2\omega\tau}}{\substack{\Re(f_0)+(\Re(f_1)+\Re(g_1))\cos\omega\tau(\Im(f_1)-\Im(g_1))\sin\omega\tau \\ +(\Re(f_2)+\Re(g_2))\cos 2\omega\tau+(\Im(f_2)-\Im(g_2))\sin 2\omega\tau}}\right) \tag{S28}$$

using the approximation $\arctan(\theta) \approx \theta$ and Taylor expansion of $\cos\omega\tau$ and $\sin\omega\tau$ gives,

$$\phi(\omega) \approx \phi_0 + \alpha\omega + \beta\omega^2 + \cdots \tag{S29}$$

β is the coefficient of the ω^2 term in the expansion and is called the spectral chirp.

$$\beta = -\frac{(\Im(f_1) + \Im(g_1))/2 + 2(\Im(f_2) + \Im(g_2)}{\substack{\Re(f_0)+(\Re(f_1)+\Re(g_1))\cos\omega\tau+(\Im(f_1)-\Im(g_1))\sin\omega\tau \\ +(\Re(f_2)+\Re(g_2))\cos 2\omega\tau+(\Im(f_2)-\Im(g_2))\sin 2\omega\tau}}\tau^2 \tag{S30}$$

In general, the relation between temporal and spectral chirp is non-trivial, but within the narrow pulse limit, the temporal chirp is proportional to the reciprocal of the spectral chirp.

$$\beta_t = \frac{1}{4\beta} = \frac{-1}{4\tau^2}\frac{\substack{\Re(f_0)+(\Re(f_1)+\Re(g_1))\cos\omega\tau+(\Im(f_1)-\Im(g_1))\sin\omega\tau \\ +(\Re(f_2)+\Re(g_2))\cos 2\omega\tau+(\Im(f_2)-\Im(g_2))\sin 2\omega\tau}}{(\Im(f_1) + \Im(g_1))/2 + 2(\Im(f_2) + \Im(g_2)} \tag{S31}$$

This shows that the temporal chirp not only has the $\cos\omega\tau$ terms, which means the temporal chirp also oscillates with fundamental/central frequency, but also contains higher frequency contributions due to $\cos 2\omega\tau$ terms.

3 Dispersion broadening in Magnesium Oxide

A transform-limited Gaussian pulse passing through a dispersive medium acquires a quadratic spectral phase. Different frequency components travel at different group velocities, which stretches

the pulse in time. For a Gaussian pulse with input FWHM duration $\delta_0 = 50$ fs, the output FWHM after propagating a distance z is:

$$\delta(z) = \delta_0 \sqrt{1 + \frac{4 \ln 2 \beta_2 z}{\delta_0^2}} \tag{S32}$$

where $\beta_2 = \frac{\partial^2 \phi}{\partial \omega^2}$ is the group delay dispersion (GDD) of the material. The GDD $\beta_2 = \frac{\lambda^3}{2\pi c^2} \frac{\partial^2 n}{\partial \lambda^2}$ can be calculated using the Sellmeier coefficients for MgO (*77*) ($\beta_2 \approx 84$ fs^2/mm). The broadening for a $\lambda = 800$ nm pulse propagating through a $z = 1$ mm thick sample is $\delta = 50.22$ fs. This shows that the dispersion due to the propagation of an 800 nm pulse in MgO is negligible.

4 Time-dependent perturbation theory with band-structure modulation

In the DFWM process, the electron transitions from the Valence band (VB) to the Conduction band (CB) in a sufficiently strong incoming electric field. The output electric field $E_{DFWM} \propto \frac{\partial \langle J(t) \rangle^{(3)}}{\partial t}$.

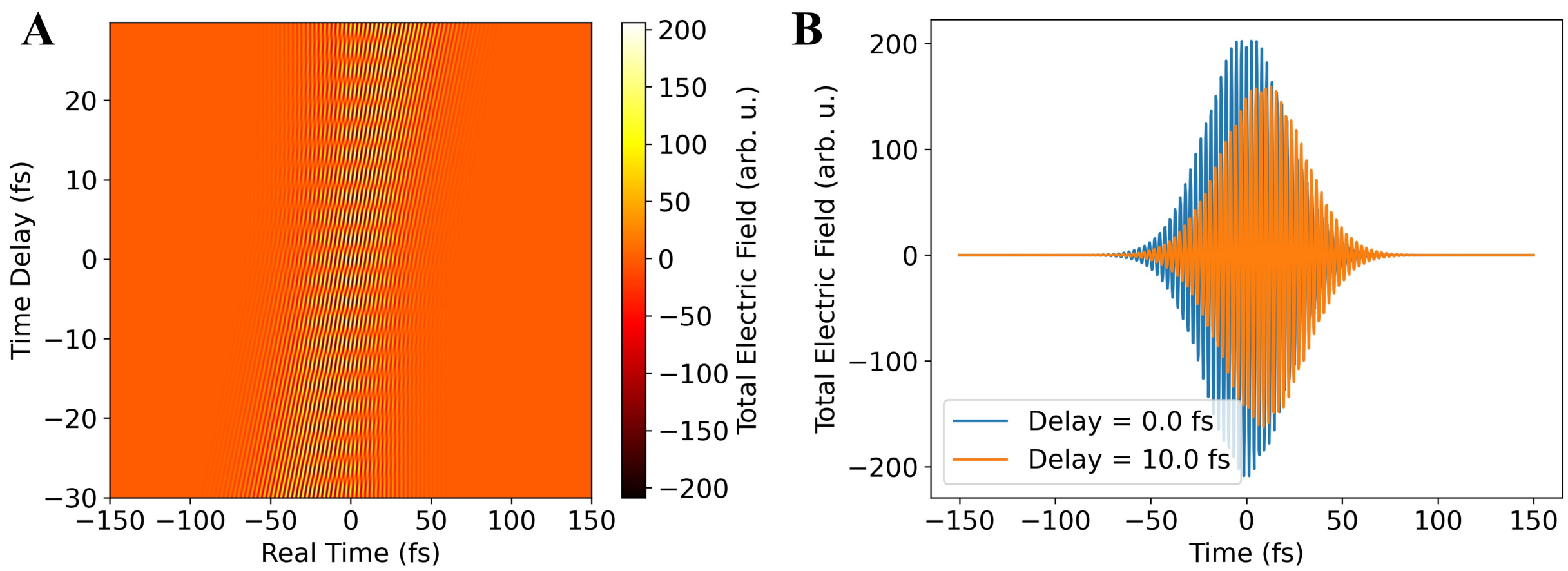

Figure S4: **Simulation of total input electric field**: ((A) The simulated total electric field ($E_1(t-\tau) + E_2(t-\tau) + E_3(t)$) as a function of real time and time delay. (B) The total electric field for $\tau = 0$ fs and $\tau = 10$ fs is shown.

A simple extension of the Kubo formalism gives us a general expression of the third-order expectation value of current (*42*),

$$\langle J \rangle^{(3)} = (-i)^3 \int_{t_0}^{t} dt_3 \int_{t_0}^{t_3} dt_2 \int_{t_0}^{t_2} dt_1 \left\langle \left[\left[\left[\hat{J}(t), \hat{V}(t_1) \right], \hat{V}(t_2) \right], \hat{V}(t_3) \right] \right\rangle_0 \tag{S33}$$

where $V(t)$ is the external potential and $\hat{O}$ represents any operator O in the interaction picture. The expectation value $\langle\rangle_0$ is with respect to the unperturbed Hamiltonian H_0. The external potential in the DFWM process is $V(t) = \int d^3\mathbf{r}\, \mathbf{J}(\mathbf{r}) \cdot \mathbf{A}_{ext}(\mathbf{r}, t)$, with $\mathbf{J}(\mathbf{r})$ being the current operator and $\mathbf{A}_{ext}$ being the vector potential of the total incoming electric field $\mathbf{E}_{ext}(\mathbf{r}, t) = \mathbf{E}_g(t-\tau)e^{i\omega_0(t-\tau)-i\mathbf{k}_1\cdot\mathbf{r}} + \mathbf{E}_g(t-\tau)e^{i\omega_0(t-\tau)-i\mathbf{k}_2\cdot\mathbf{r}} + \mathbf{E}_p(t)e^{i\omega_0 t-i\mathbf{k}_3\cdot\mathbf{r}} + c.c.$, where τ is the time delay between Probe and Gate

pulses. Plugging all the terms into Eq S33,

$$\begin{aligned}\langle J^i(t)\rangle^{(3)} = \frac{(-i)^3}{\sqrt{A}} \int_{t_0}^{t} dt_3 \int_{t_0}^{t_3} dt_2 \int_{t_0}^{t_2} dt_1\ A^{\alpha}_{ext}(t_1)A^{\beta}_{ext}(t_2)A^{\gamma}_{ext}(t_3) \\ \times \underbrace{j^i_{m,n,\mathbf{q}} j^{\alpha}_{m_1,n_1,\mathbf{q}_1} j^{\beta}_{m_2,n_2,\mathbf{q}_2} j^{\gamma}_{m_3,n_3,\mathbf{q}_3}}_{\text{time-independent terms}} \\ \times \exp\left(i\Omega_{m,n,\mathbf{q}}t + i\Omega_{m_1,n_1,\mathbf{q}_1}t_1 + i\Omega_{m_2,n_2,\mathbf{q}_2}t_2 + i\Omega_{m_3,n_3,\mathbf{q}_3}t_3\right) \\ \times \underbrace{\left\langle \left[\left[\left[a^{\dagger}_{m,\mathbf{q}}a_{n,\mathbf{q}}, a^{\dagger}_{m_1,\mathbf{q}_1}a_{n_1,\mathbf{q}_1}\right], a^{\dagger}_{m_2,\mathbf{q}_2}a_{n_2,\mathbf{q}_2}\right], a^{\dagger}_{m_3,\mathbf{q}_3}a_{n_3,\mathbf{q}_3}\right]\right\rangle_0}_{=O(\text{Operator part})}\end{aligned} \quad \text{(S34)}$$

Here, $\Omega_{m,n,\mathbf{q}}$ represents the energy difference between the mth and n^{th} bands and t_0 is the initial time when there is no interaction (In most cases, $t_0 = -\infty$ indicating no perturbation at time $-\infty$). For brevity, Einstein's summation convention is applied to all indices in the last expression. For the remainder of the calculation, we assume that the momentum carried by the photons ($\sim 2\pi/\lambda$) is an order of magnitude smaller than the crystal momentum of the electrons ($\sim 2\pi/a$, a is the lattice parameter of MgO). Atomic units are used throughout these derivations. Thus, photon-assisted electron excitation results in a negligible change in crystal momentum $\mathbf{k}$ (The change would be significant if we included the effects of phonons, which are not considered in this model). The $j^i_{m,n,\mathbf{q}}$ represents the matrix elements of the current operators and is proportional to the transition dipole matrix elements between the valence band (VB) and the conduction band (CB). Since the current matrix element (transition-dipole moment) varies weakly as a function of momentum k (*8, 13*), it can be considered a constant and absorbed into the normalization constant. The electronic band structure of MgO was calculated using density functional theory (DFT) within the generalized gradient approximation (GGA) employing the Perdew–Burke–Ernzerhof (PBE) functional for exchange–correlation effects. It is well established that the PBE functional tends to underestimate the band gap in wide-bandgap insulators (*78*). Therefore, the calculated band gap of approximately 5 eV was corrected to the experimental value of 7.8 eV using the scissors approximation (*79*).

The operator part in Eq. S34 can be solved using the anti-commutation relation of fermions. The appropriate non-zero terms that satisfy the time ordering condition $t_1 < t_2 < t_3$ are chosen. In general, converging all orders of the response function requires considering multiple conduction

bands (*44*). Since we are only interested in the third-order response, it turns out that only two bands are sufficient to describe the off-resonant DFWM interaction in MgO ($\hbar\omega_0 = 1.55eV < \Delta_{\mathrm{gap}} = 7.78eV$). In addition, the intensity of the IR pulses in the experiment is not large enough for the higher-order multiphoton absorption process to contribute to the lower-order response function (*44*). Thus, $O \sim \langle a_m^\dagger a_m \rangle - \langle a_n^\dagger a_n \rangle \approx 1$ if $m \neq n$ for a two-band system.

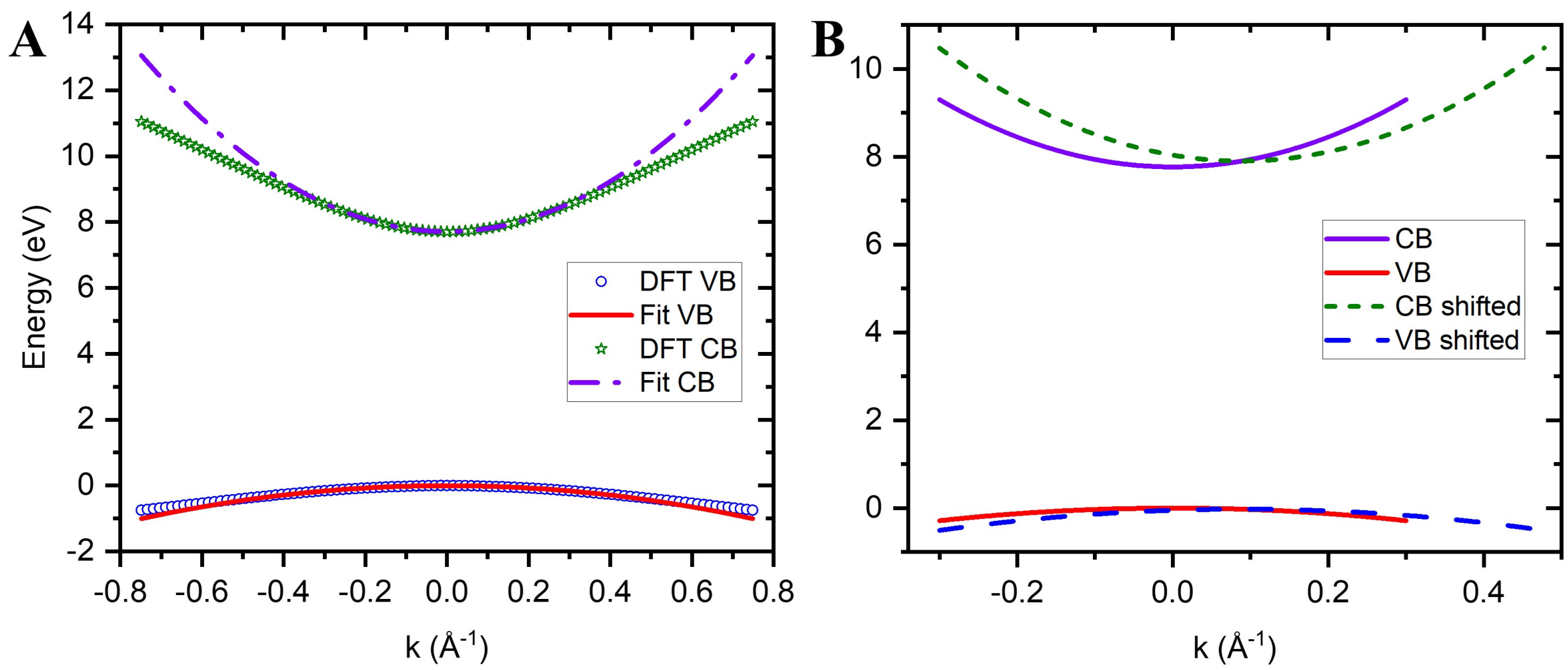

Figure S5: **Shift in the Band structure**: Panel (A) presents the valence band (VB) and conduction band (CB) structure of MgO, calculated using density functional theory (DFT; see main text for details), with blue circles and green stars as markers, respectively. The parabolic band fitting procedure is applied to the calculated band structure to extract the effective masses: $m^*_{CB} = 0.4\ m_e$ and $m^*_{VB} = 2.13\ m_e$. Panel (B) displays the shifted VB (blue, dashed curve) and CB (green, dashed curve), evaluated at $t = 1$ fs and $\tau = 0$ fs.

According to the Bloch acceleration theorem, the single-particle states become time dependent in reciprocal space. The crystal momentum transforms as $\mathbf{p}(t) = \mathbf{p}_0 - e \int_{t_0}^{t} \mathbf{E}_{ext}(t')dt'$, where $\mathbf{E}_{ext}(t)$ is the total input electric field. The simulated total input electric field as a function of time and time delay is shown in Fig. S4. Thus, the valence and conduction band energies will also be modified as $\epsilon_i(t) = \epsilon_i \left(\mathbf{p}_0 - e \int_{t_0}^{t} \mathbf{E}_{ext}(t')dt' \right)$ with $i = \{CB, VB\}$.

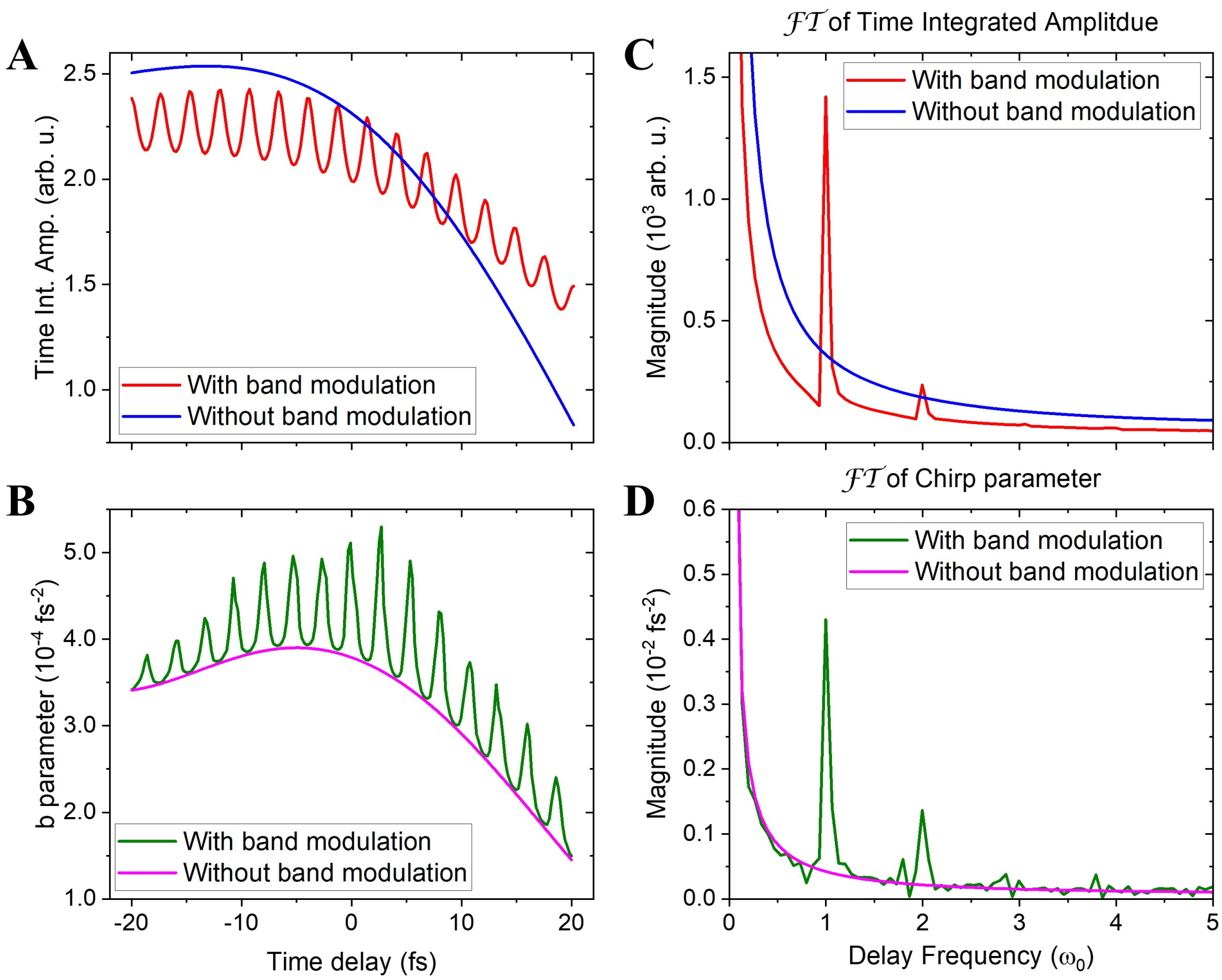

Figure S6: **Field observables with and without band structure modulation**: (A) Time-integrated amplitude and (B) temporal chirp are calculated using time-dependent perturbation theory with and without band structure modulation. Their corresponding Fourier transformations in the time delay domain are shown in (C) and (D), respectively.

Eq S34 is then simplified to get,

$$\langle J^i(t)\rangle^{(3)} = \mathcal{N}_{\mathbf{q}}^{i\alpha\beta\gamma} \int_{t_0}^{t} dt_3 \int_{t_0}^{t_3} dt_2 \int_{t_0}^{t_2} dt_1 \; A_{ext}^{\alpha}(t_1) A_{ext}^{\beta}(t_2) A_{ext}^{\gamma}(t_3)$$
$$\times \exp\left(i \int_{t_0}^{t} \Theta_{0,1,\mathbf{q}}(t')dt' + i \int_{t_0}^{t_1} \Theta_{1,0,\mathbf{q}}(t')dt' + i \int_{t_0}^{t_2} \Theta_{0,1,\mathbf{q}}(t')dt' + i \int_{t_0}^{t_3} \Theta_{1,0,\mathbf{q}}(t')dt'\right) \quad \text{(S35)}$$

where, $\Theta_{1,0,\mathbf{q}}(t)$ is the time-dependent energy gap and is defined by $\Theta_{1,0,\mathbf{q}}(t) = \epsilon_{CB}(t) - \epsilon_{VB}(t)$. Also, 0, 1 corresponds to VB, CB respectively for the two-band model.

The $E_g(t)$ and $E_p(t)$ are the envelope functions for the Gate and Probe pulses, which are adopted from the experimental data (*27*). Since the Keldysh parameter for our experiment is large ($>\sim 48$),

the band modification is much smaller than in the case of a small Keldysh parameter (*36, 30*). Thus, for numerical convenience, we assume the parabolic band approximation, i.e. $\epsilon_{VB} \approx -|\mathbf{p}|^2/2m_{VB}$ and $\epsilon_{CB} \approx \Delta\left(1 + |\mathbf{p}|^2/(2m_{CB}\Delta)\right)$. The effective masses m_{VB} and m_{CB} are extracted by fitting to the calculated band structure. Fig. S5 A presents the parabolic bands fitted to those obtained from density functional theory (DFT) calculations. The strong-field interaction induces shifts in the valence band (VB) and conduction band (CB), as illustrated in Fig. S5 B, resulting in a change in the bandgap ($\delta\Delta \sim 1$ eV).

Lastly, the signal was measured along the direction of $\mathbf{k}_1 - \mathbf{k}_2 + \mathbf{k}_3$ in the experiment. Since $\mathbf{A}_{ext}(t)$ is composed of six terms (3 + 3 cc), Eq. S35 will have contributions from 216 terms. Of these, only 12 (6 + 6 cc) of the terms contribute to the phase matching condition of $\mathbf{k}_1 - \mathbf{k}_2 + \mathbf{k}_3$.

In Fig. S6 A and B, we show that the time-integrated amplitude and temporal chirp parameter respectively, calculated using time-dependent perturbation theory without the inclusion of band modulation (blue/magenta curves, see Eq. (8) of the main text) and after explicitly including the band structure modulation (red/green curves, see Eq. (9) of the main text). Fig. S6 C and D are the Fourier transformation of A and B, respectively. This shows that the peaks we see in the experimentally measured field observables in Fig. (3) are due to the band structure modulation of MgO as it interacts with the Gate and Probe pulses.

5 Semiconductor Bloch Equation

To fully understand strong field–matter interactions, we employ a non-perturbative microscopic model based on a one-dimensional two-band representation of MgO. When the crystal interacts with a strong laser field $E(t)$, electrons are driven from the valence band to the conduction band, the transition strength being determined by the element of the transition dipole matrix

$$d_k^{cv} = \frac{1}{ia_0}\int u_{c,k}^{*}\frac{\partial}{\partial k}u_{v,k}\,dx, \tag{S36}$$

where $u_{n,k}$ are the cell-periodic parts of the Bloch functions and a_0 is the lattice constant (*9*).

The transient electron dynamics under a strong, time-dependent electric field are described using the semiconductor Bloch equations (SBEs), which govern the evolution of the electron (hole)

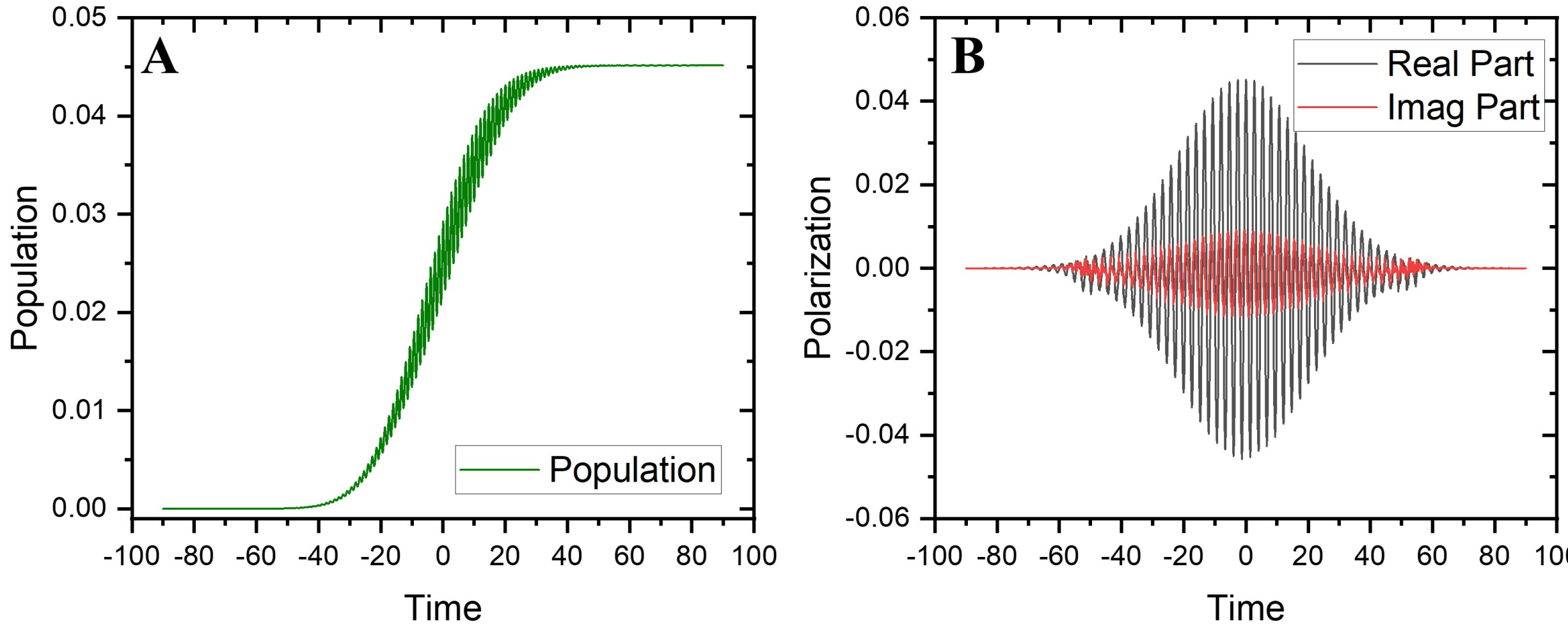

Figure S7: **Simulated population and polarization calculated using SBE**: (A) Evolution of the microscopic population ($n_k^e(t)$) under excitation by the incoming electric field. (B) Real (black) and imaginary (red) components of the time-dependent microscopic polarization ($p_k^e(t)$) are presented.

populations n_k^e (n_k^h) and the microscopic polarization $p_k^{h,e}$ between them (*12, 13, 14*):

$$\hbar \frac{\partial n_k^e}{\partial t} = -2\hbar \Im \left[d_k^{cv} E(t) \right] + |e| E(t) \nabla_k n_k^e - \frac{1}{2T_1} (n_k^e - n_{-k}^e), \tag{S37a}$$

$$\hbar \frac{\partial n_k^h}{\partial t} = -2\hbar \Im \left[d_k^{cv*} E(t) \right] + |e| E(t) \nabla_k n_k^h - \frac{1}{2T_1} (n_k^h - n_{-k}^h), \tag{S37b}$$

$$i\hbar \frac{\partial p_k^{h,e}}{\partial t} = \left(\epsilon_k^e + \epsilon_k^h - i\frac{\hbar}{T_2} \right) p_k^{h,e} - d_k^{cv} E(t) \left(1 - n_k^e - n_k^h \right) + i|e| E(t) \nabla_k p_k^{h,e}. \tag{S37c}$$

Here, ϵ_k^e and ϵ_k^h denote the electron and hole dispersion relations, respectively, defined as $\epsilon_k^e = \epsilon_k^c$ and $\epsilon_k^h = -\epsilon_k^v$, where ϵ_k^c and ϵ_k^v correspond to the conduction and valence band energies. The phenomenological relaxation and dephasing times are set to $T_1 = 7$ fs and $T_2 = 1.1$ fs (*13, 14*). Fig. S7 A and B show the time evolution of population $n_k^e(t)$ and microscopic polarization $p_k^{h,e}(t)$ at the Γ point ($k = 0$) of the MgO. The input electric field parameters corresponding to the experiment are used in these calculations. From the time-dependent evolution of the electron (hole) populations and the microscopic polarization, we calculate the intraband and interband currents, which serve as the primary current sources arising from the acceleration of excited carrier wavepackets within the bands and the temporal variation of the polarization between electron–hole pairs, respectively (*14*).

$$J_{\text{intra}}(t) = \frac{e}{\hbar} \sum_{k,i} \nabla_k \epsilon_k^i n_k^i \tag{S38a}$$

$$P_{\text{inter}}(t) = \sum_k d_k^{cv} p_k^{h,e} \tag{S38b}$$

where $i = \{e, h\}$. Both currents will contribute to the total ouput electric field $E_{\text{out}}(t) \propto J_{\text{intra}}(t) + \frac{\partial}{\partial t} P_{\text{inter}}(t)$.

6 Evidence Supporting Model-Experiment Agreement

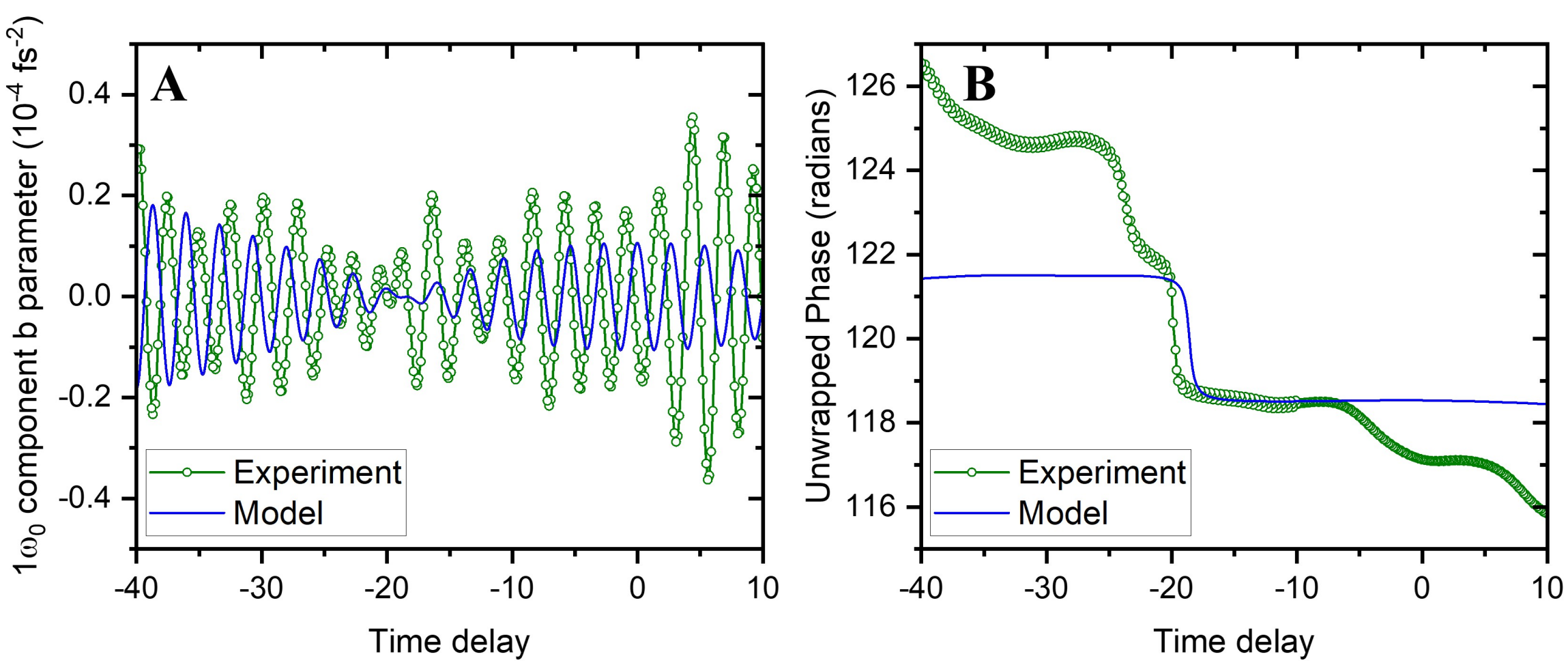

Figure S8: The $1\omega_0$ component extracted from the Fourier transformation of the temporal chip is isolated, and the inverse Fourier transformation is performed. (A) The oscillation of the $1\omega_0$ component of the chirp parameter extracted from the experimentally measured DFWM electric field (green) is compared with the nonlinear susceptibility modulation model (magenta) as a function of time delay. (B) The time-dependent unwrapped phases of the oscillations in (A) are also presented, showing that the susceptibility modulation model accurately reproduces the π-phase jump near $\tau = -20$ fs, consistent with the experimental observations.

To further support this interpretation, we isolate the $1\omega_0$ peak of the measured chirp in Fig. 3 B and inverse Fourier transform to obtain the single frequency filtered oscillation of the temporal chirp, shown in Fig. S8 A. This is then compared with the $1\omega_0$ component of the chirp calculated

using Eq. S25. Fig. S8 B shows the time delay-dependent phase of the oscillations in Fig. S8 A. The model accurately reproduces the π-phase jump in the chirp oscillation near $\tau = -20$ fs, in excellent agreement with the experimental observation, as shown in Fig. S8 B. The offset of this phase jump from zero time delay is due to the chirp present in the input fields.

REFERENCES

1. S. Ghimire, A. D. DiChiara, E. Sistrunk, P. Agostini, L. F. DiMauro, D. A. Reis, Observation of high-order harmonic generation in a bulk crystal. *Nat. Phys.* **7**, 138–141 (2011).

2. S. Ghimire, D. A. Reis, High-harmonic generation from solids. *Nat. Phys.* **15**, 10–16 (2019).

3. S. Mitra, Á. Jiménez-Galán, M. Aulich, M. Neuhaus, R. E. F. Silva, V. Pervak, M. F. Kling, S. Biswas, Light-wave-controlled Haldane model in monolayer hexagonal boron nitride. *Nature* **628**, 752–757 (2024).

4. A. P. Jauho, K. Johnsen, Dynamical Franz-Keldysh effect. *Phys. Rev. Lett.* **76**, 4576–4579 (1996).

5. K. B. Nordstrom, K. Johnsen, S. J. Allen, A.-P. Jauho, B. Birnir, J. Kono, T. Noda, H. Akiyama, H. Sakaki, Excitonic dynamical Franz-Keldysh effect. *Phys. Rev. Lett.* **81**, 457–460 (1998).

6. M. Lucchini, S. A. Sato, A. Ludwig, J. Herrmann, M. Volkov, L. Kasmi, Y. Shinohara, K. Yabana, L. Gallmann, U. Keller, Attosecond dynamical Franz-Keldysh effect in polycrystalline diamond. *Science* **353**, 916–919 (2016).

7. J. Reislöhner, X. Chen, D. Kim, S. Botti, A. N. Pfeiffer, Dynamical Franz-Keldysh effect in diamond in the deep ultraviolet probed by transient absorption and dispersion spectroscopy using a miniature beamline. *Phys. Rev. Lett.* **131**, 136902 (2023).

8. G. Vampa, C. R. McDonald, G. Orlando, D. D. Klug, P. B. Corkum, T. Brabec, Theoretical analysis of high-harmonic generation in solids. *Phys. Rev. Lett.* **113**, 073901 (2014).

9. M. Wu, S. Ghimire, D. A. Reis, K. J. Schafer, M. B. Gaarde, High-harmonic generation from Bloch electrons in solids. *Phys. Rev. A* **91**, 043839 (2015).

10. E. N. Osika, A. Chacón, L. Ortmann, N. Suárez, J. A. Pérez-Hernández, B. Szafran, M. F. Ciappina, F. Sols, A. S. Landsman, M. Lewenstein, Wannier-Bloch approach to localization in high-harmonics generation in solids. *Phys. Rev. X* **7**, 021017 (2017).

11. R. E. F. Silva, F. Martín, M. Ivanov, High harmonic generation in crystals using maximally localized Wannier functions. *Phys. Rev. B* **100**, 195201 (2019).

12. O. Schubert, M. Hohenleutner, F. Langer, B. Urbanek, C. Lange, U. Huttner, D. Golde, T. Meier, M. Kira, S. W. Koch, R. Huber, Sub-cycle control of terahertz high-harmonic generation by dynamical Bloch oscillations. *Nat. Photonics* **8**, 119–123 (2014).

13. M. Hohenleutner, F. Langer, O. Schubert, M. Knorr, U. Huttner, S. W. Koch, M. Kira, R. Huber, Real-time observation of interfering crystal electrons in high-harmonic generation. *Nature* **523**, 572–575 (2015).

14. U. Huttner, M. Kira, S. W. Koch, Ultrahigh off-resonant field effects in semiconductors. *Laser Photon. Rev.* **11**, 1700049 (2017).

15. T. Otobe, Y. Shinohara, S. A. Sato, K. Yabana, Femtosecond time-resolved dynamical Franz-Keldysh effect. *Phys. Rev. B* **93**, 045124 (2016).

16. R. W. Boyd, *Nonlinear Optics* (Academic Press Inc., ed. 3, 2008).

17. D. A. B. Miller, D. S. Chemla, D. J. Eilenberger, P. W. Smith, A. C. Gossard, W. Wiegmann, Degenerate four-wave mixing in room-temperature GaAs/GaAlAs multiple quantum well structures. *Appl. Phys. Lett.* **42**, 925–927 (1983).

18. H. Nickolaus, F. Henneberger, Biexcitonic four-wave-mixing signal in quantum wells: Photon-echo versus free-induction decay. *Phys. Rev. B* **57**, 8774–8777 (1998).

19. M. Dignam, M. Hawton, Intraband polarization as the source of degenerate four-wave mixing signals in asymmetric semiconductor quantum well structures. *Phys. Rev. B* **67**, 035329 (2003).

20. A. H. Flayyih, A. H. Al-Khursan, Theory of four-wave mixing in quantum dot semiconductor optical amplifiers. *J. Phys. D Appl. Phys.* **46**, 445102 (2013).

21. S. Yu, J. I. Lee, A. K. Viswanath, Time-resolved four-wave mixing signal in thick bulk GaAs. *J. Appl. Phys.* **86**, 3159–3164 (1999).

22. A. Schulze, A. Knorr, S. W. Koch, Pulse propagation and many-body effects in semiconductor four-wave mixing. *Phys. Rev. B* **51**, 10601–10609 (1995).

23. P. Schillak, I. Balslev, Theory of propagation effects in time-resolved four-wave mixing. *Phys. Rev. B* **48**, 9426–9433 (1993).

24. F. Bencivenga, R. Cucini, F. Capotondi, A. Battistoni, R. Mincigrucci, E. Giangrisostomi, A. Gessini, M. Manfredda, I. P. Nikolov, E. Pedersoli, E. Principi, C. Svetina, P. Parisse, F. Casolari, M. B. Danailov, M. Kiskinova, C. Masciovecchio, Four-wave mixing experiments with extreme ultraviolet transient gratings. *Nature* **520**, 205–208 (2015).

25. L. Foglia, F. Capotondi, R. Mincigrucci, D. Naumenko, E. Pedersoli, A. Simoncig, G. Kurdi, A. Calvi, M. Manfredda, L. Raimondi, N. Mahne, M. Zangrando, C. Masciovecchio, F. Bencivenga, First evidence of purely extreme-ultraviolet four-wave mixing. *Phys. Rev. Lett.* **120**, 263901 (2018).

26. S. Han, L. Ortmann, H. Kim, Y. W. Kim, T. Oka, A. Chacon, B. Doran, M. Ciappina, M. Lewenstein, S.-W. Kim, S. Kim, A. S. Landsman, Extraction of higher-order nonlinear electronic response in solids using high harmonic generation. *Nat. Commun.* **10**, 3272 (2019).

27. F. Walz, S. Pandey, L. Z. Tan, N. Shivaram, Electric field measurement of femtosecond time-resolved four-wave mixing signals in molecules. *Opt. Express* **30**, 36065–36072 (2022).

28. S. Pandey, L. Z. Tan, F. Walz, V. Makhija, N. Shivaram, Ultrafast temporal phase-resolved nonlinear optical spectroscopy in the molecular frame. *Optica* **11**, 776–781 (2024).

29. D. N. Fittinghoff, J. L. Bowie, J. N. Sweetser, R. T. Jennings, M. A. Krumbügel, K. W. DeLong, R. Trebino, I. A. Walmsley, Measurement of the intensity and phase of ultraweak, ultrashort laser pulses. *Opt. Lett.* **21**, 884–886 (1996).

30. V. Gruzdev, O. Sergaeva, Ultrafast modification of band structure of wide-band-gap solids by ultrashort pulses of laser-driven electron oscillations. *Phys. Rev. B* **98**, 115202 (2018).

31. M. Yeung, A. von Hoegen, E. Viñas Boström, F. Zhao, F. Ritzkowsky, J. B. Maier, G. L. Dolso, C. Heide, D. G. Chica, X. Roy, K. K. Berggren, A. Rubio, P. D. Keathley, N. Gedik,

Field-resolved observation of exciton coherence in a van der Waals magnet. *Nat. Mater.* (2026).

32. J. A. Shirley, R. J. Hall, A. C. Eckbreth, Folded BOXCARS for rotational Raman studies. *Opt. Lett.* **5**, 380–382 (1980).

33. A. H. Chin, O. G. Calderón, J. Kono, Extreme midinfrared nonlinear optics in semiconductors. *Phys. Rev. Lett.* **86**, 3292–3295 (2001).

34. A. J. Uzan, G. Orenstein, Á. Jiménez-Galán, C. McDonald, R. E. Silva, B. D. Bruner, N. D. Klimkin, V. Blanchet, T. Arusi-Parpar, M. Krüger, N. Dudovich, Attosecond spectral singularities in solid-state high-harmonic generation. *Nat. Photonics* **14**, 183–187 (2020).

35. A. J. Uzan-Narovlansky, Á. Jiménez-Galán, G. Orenstein, R. E. Silva, T. Arusi-Parpar, S. Shames, B. D. Bruner, B. Yan, O. Smirnova, M. Ivanov, N. Dudovich, Observation of light-driven band structure via multiband high-harmonic spectroscopy. *Nat. Photonics* **16**, 428–432 (2022).

36. O. Neufeld, W. Mao, H. Hübener, N. Tancogne-Dejean, S. A. Sato, U. De Giovannini, A. Rubio, Time- and angle-resolved photoelectron spectroscopy of strong-field light-dressed solids: Prevalence of the adiabatic band picture. *Phys. Rev. Res.* **4**, 033101 (2022).

37. F. Wang, W. Chen, C. Bao, T. Lin, H. Zhong, H. Zhang, S. Zhou, Light-field dressing of transient photoexcited states above the Fermi energy. *Phys. Rev. Lett.* **134**, 146401 (2025).

38. V. Tiwari, B. Gu, I. Franco, Floquet theory and computational method for the optical absorption of laser-dressed solids. *Phys. Rev. B* **108**, 064308 (2023).

39. K.-H. Hong, J. Kim, S.-H. Lee, J. K. Shin, Strain-driven electronic band structure modulation of Si nanowires. *Nano Lett.* **8**, 1335–1340 (2008).

40. Y. Han, J. Fang, X. Xing, H. Gao, J. Zhang, Y. Zhang, Y. Yuan, J. Yang, S. Li, Z. Shi, Band structure modulation of chalcogenide perovskite with Eu as A-site cation. *Appl. Phys. Lett.* **126**, 081903 (2025).

41. L. E. Sánchez-González, M. A. Mojarro, J. A. Maytorena, R. Carrillo-Bastos, Band structure and optical response of Kekulé-modulated $\alpha - T_3$ model. *Phys. Rev. B* **111**, 115417 (2025).

42. H. Bruus, K. Flensberg, *Many-Body Quantum Theory in Condensed Matter Physics* (OUP Oxford, 2004).

43. L. Keldysh, The effect of a strong electric field on the optical properties of insulating crystals. *Sov. Phys. JETP* **7**, 788–790 (1958).

44. D. Popova-Gorelova, R. Santra, Microscopic nonlinear optical response: Analysis and calculations with the Floquet-Bloch formalism. *Struct. Dyn.* **11**, 014102 (2024).

45. J. Wilhelm, P. Grössing, A. Seith, J. Crewse, M. Nitsch, L. Weigl, C. Schmid, F. Evers, Semiconductor Bloch-equations formalism: Derivation and application to high-harmonic generation from Dirac fermions. *Phys. Rev. B* **103**, 125419 (2021).

46. L. Yue, M. B. Gaarde, Structure gauges and laser gauges for the semiconductor Bloch equations in high-order harmonic generation in solids. *Phys. Rev. A* **101**, 053411 (2020).

47. A. Sommer, E. M. Bothschafter, S. A. Sato, C. Jakubeit, T. Latka, O. Razskazovskaya, H. Fattahi, M. Jobst, W. Schweinberger, V. Shirvanyan, V. S. Yakovlev, R. Kienberger, K. Yabana, N. Karpowicz, M. Schultze, F. Krausz, Attosecond nonlinear polarization and light–matter energy transfer in solids. *Nature* **534**, 86–90 (2016).

48. R. Stolen, A. Ashkin, Optical Kerr effect in glass waveguide. *Appl. Phys. Lett.* **22**, 294–296 (1973).

49. D. N. Schimpf, T. Eidam, E. Seise, S. Hädrich, J. Limpert, A. Tünnermann, Circular versus linear polarization in laser-amplifiers with Kerr-nonlinearity. *Opt. Express* **17**, 18774–18781 (2009).

50. C. Brée, A. Demircan, G. Steinmeyer, Saturation of the all-optical Kerr effect. *Phys. Rev. Lett.* **106**, 183902 (2011).

51. A. Widhalm, C. Golla, N. Weber, P. Mackwitz, A. Zrenner, C. Meier, Electric-field-induced second harmonic generation in silicon dioxide. *Opt. Express* **30**, 4867–4874 (2022).

52. R. Yanagimoto, B. A. Ash, M. M. Sohoni, M. M. Stein, Y. Zhao, F. Presutti, M. Jankowski, L. G. Wright, T. Onodera, P. L. McMahon, Programmable on-chip nonlinear photonics. *Nature* **649**, 330–337 (2026).

53. M. Schultze, E. M. Bothschafter, A. Sommer, S. Holzner, W. Schweinberger, M. Fiess, M. Hofstetter, R. Kienberger, V. Apalkov, V. S. Yakovlev, M. I. Stockman, F. Krausz, Controlling dielectrics with the electric field of light. *Nature* **493**, 75–78 (2013).

54. Y. Kim, M. J. Kim, S. Cha, S. Choi, C.-J. Kim, B. J. Kim, M.-H. Jo, J. Kim, J. Lee, Dephasing dynamics accessed by high harmonic generation: Determination of electron–hole decoherence of Dirac fermions. *Nano Lett.* **24**, 1277–1283 (2024).

55. S. Mokhtari, V. Jelic, D. N. Purschke, S. Gholam-Mirzaei, K. M. Kowalczyk, D. A. Reis, T. J. Hammond, D. M. Villeneuve, A. Staudte, F. Légaré, G. Vampa, Phonon-driven decoherence of high-harmonic generation in the solid-state. arXiv:2604.07543 [cond-mat.mes-hall] (2026).

56. T. L. Purz, B. T. Hipsley, E. W. Martin, R. Ulbricht, S. T. Cundiff, Rapid multiplex ultrafast nonlinear microscopy for material characterization. *Opt. Express* **30**, 45008–45019 (2022).

57. J. A. Gruetzmacher, R. A. Nome, A. M. Moran, N. F. Scherer, Assessing the dephasing dynamics of water from linear field-resolved pulse propagation experiments and simulations in highly absorbing solutions. *J. Chem. Phys.* **129**, 224502 (2008).

58. M. A. Hershberger, A. M. Moran, N. F. Scherer, New insights into response functions of liquids by electric field-resolved polarization emission time measurements. *J. Phys. Chem. B.* **115**, 5617–5624 (2011).

59. G. Orlando, T.-S. Ho, S.-I. Chu, Simple model of dephasing for high-order harmonic generation in solids. *J. Opt. Soc. Am. B* **37**, 1540 (2020).

60. M. Kolesik, J. V. Moloney, Numerical discreteness and dephasing in high-harmonic calculations in solids. *Phys. Rev. B* **108**, 115433 (2023).

61. S. Mukamel, *Principles of Nonlinear Optical Spectroscopy* (Oxford Univ. Press, 1995).

62. G. G. Brown, A. Jiménez-Galán, R. E. F. Silva, M. Ivanov, Real-space perspective on dephasing in solid-state high harmonic generation. *Phys. Rev. Res.* **6**, 043005 (2024).

63. L.-M. Koll, S. V. B. Jensen, P. J. van Essen, B. de Keijzer, E. Olsson, J. Cottom, T. Witting, A. Husakou, M. J. J. Vrakking, L. B. Madsen, P. M. Kraus, P. Jürgens, Extreme ultraviolet high-harmonic interferometry of excitation-induced bandgap dynamics in solids. *Optica* **12**, 1606–1614 (2025).

64. D. Ceus, F. Reynaud, J. Woillez, O. Lai, L. Delage, L. Grossard, R. Baudoin, J.-T. Gomes, L. Bouyeron, H. Herrmann, W. Sohler, Application of frequency conversion of starlight to high-resolution imaging interferometry. On-sky sensitivity test of a single arm of the interferometer. *Mon. Not. R. Astron. Soc. Lett.* **427**, L95–L98 (2012).

65. R. S. Bondurant, P. Kumar, J. H. Shapiro, M. Maeda, Degenerate four-wave mixing as a possible source of squeezed-state light. *Phys. Rev. A* **30**, 343–353 (1984).

66. G. Breitenbach, S. Schiller, J. Mlynek, Measurement of the quantum states of squeezed light. *Nature* **387**, 471–475 (1997).

67. H. H. Adamyan, J. A. Bergou, N. T. Gevorgyan, G. Y. Kryuchkyan, Strong squeezing in periodically modulated optical parametric oscillators. *Phys. Rev. A* **92**, 053818 (2015).

68. T. Onodera, E. Ng, C. Gustin, N. Lörch, A. Yamamura, R. Hamerly, P. L. McMahon, A. Marandi, H. Mabuchi, Nonlinear quantum behavior of ultrashort-pulse optical parametric oscillators. *Phys. Rev. A* **105**, 033508 (2022).

69. C. Fabre, N. Treps, Modes and states in quantum optics. *Rev. Mod. Phys.* **92**, 035005 (2020).

70. C. Riek, P. Sulzer, M. Seeger, A. S. Moskalenko, G. Burkard, D. V. Seletskiy, A. Leitenstorfer, Subcycle quantum electrodynamics. *Nature* **541**, 376–379 (2017).

71. M. Sennary, J. Rivera-Dean, M. ElKabbash, V. Pervak, M. Lewenstein, M. T. Hassan, Attosecond quantum uncertainty dynamics and ultrafast squeezed light for quantum communication. *Light Sci. Appl.* **14**, 350 (2025).

72. R. Zimmerman, S. Kumar, S. K. Tiwari, E. Liu, F. Walz, S. Pandey, G. J. Economou, H. Alaeian, C.-T. Liao, V. Walther, N. Shivaram, Attosecond control of squeezed light. arXiv:2512.17046 [quant-ph] (2025).

73. H. Vahlbruch, M. Mehmet, K. Danzmann, R. Schnabel, Detection of 15 dB squeezed states of light and their application for the absolute calibration of photoelectric quantum efficiency. *Phys. Rev. Lett.* **117**, 110801 (2016).

74. R. Trebino, D. J. Kane, Using phase retrieval to measure the intensity and phase of ultrashort pulses: Frequency-resolved optical gating. *J. Opt. Soc. Am. A* **10**, 1101–1111 (1993).

75. A. Srivastava, A. Herbst, M. M. Bidhendi, M. Kieker, F. Tani, H. Fattahi, Near-petahertz fieldoscopy of liquid. *Nat. Photonics* **18**, 1320–1326 (2024).

76. D. Inukai, T. Koyama, K. Kawahara, H. Ago, H. Kishida, Complex third-order nonlinear susceptibility of single-layer graphene governing third-harmonic generation. *Phys. Rev. B* **108**, 075408 (2023).

77. R. Stephens, I. Malitson, Index of refraction of magnesium oxide. *J. Res. Natl. Bur. Stand.* **49**, 249 (1952).

78. J. M. Crowley, J. Tahir-Kheli, W. A. Goddard III, Resolution of the band gap prediction problem for materials design. *J. Phys. Chem. Lett.* **7**, 1198–1203 (2016).

79. Z. H. Levine, D. C. Allan, Linear optical response in silicon and germanium including self-energy effects. *Phys. Rev. Lett.* **63**, 1719–1722 (1989).
